# Preconception One‐Carbon Metabolism Nutrient Levels in Preparing for Pregnancy Couples and Spontaneous Pregnancy Loss: A Prospective Cohort Study

**DOI:** 10.1002/mco2.70711

**Published:** 2026-03-30

**Authors:** Xiaotian Chen, Yi Zhang, Qing Yang, Hong Zhu, Jianwei Hu, Jian Huang, Longmei Jin, Xiaohua Zhang, Yalan Dou, Wennan He, Yuanchen He, Hongyan Chen, Qinyu Yao, Yuanzhou Peng, Xiaojing Ma, Wei Sheng, Guoying Huang, Weili Yan, the SPCC group

**Affiliations:** ^1^ Department of Clinical Epidemiology & Clinical Trial Unit, National Children's Medical Center Children's Hospital of Fudan University Shanghai China; ^2^ The Maternal and Child Healthcare Institute of Songjiang District Shanghai China; ^3^ The Maternal and Child Healthcare Institute of Kunshan City Kunshan China; ^4^ Minhang Maternal and Child Health Hospital Shanghai China; ^5^ Shanghai Key Laboratory of Birth Defects, National Children's Medical Center Children's Hospital of Fudan University Shanghai China; ^6^ Pediatric Heart Center, National Children's Medical Center Children's Hospital of Fudan University Shanghai China; ^7^ Research Unit of Early Intervention of Genetically Related Childhood Cardiovascular Diseases (2018RU002) Chinese Academy of Medical Sciences Shanghai China

**Keywords:** cohort study, metabolite, one‐carbon metabolism, preconception, red blood cell folate, spontaneous pregnancy loss

## Abstract

Spontaneous pregnancy loss (SPL) remains an important yet poorly understood pregnancy outcome. One‐carbon metabolism (OCM) nutrients play an essential role in embryonic development, but their relationship with SPL remains unclear. In this prospective cohort of 11,033 couples and 2862 unpaired mothers, we assessed associations between preconception parental one‐carbon metabolism (OCM) nutrients and SPL risk. We used generalized linear models to estimate risk ratios (RRs) and 95% confidence intervals (CIs) for fathers, mothers, and the combined parental population, respectively. Each 100 ng/mL increase in paternal and maternal red blood cell (RBC) folate was associated with a 19% (aRR = 0.81; 95% CI, 0.73–0.90) and an 8% (aRR = 0.92; 0.85–0.98) lower SPL risk, respectively. The risk was reduced by 64% (aRR = 0.36; 0.16–0.79) when both parents achieved levels ≥ 400 ng/mL, compared to neither. Exploratory case–control analysis suggested associations of parental serum betaine with increased risk of SPL (β [standard error]: 0.09 [0.11] for fathers; 0.02 [0.08] for mothers) and inverse associations for taurine (−0.09 [0.11] and −0.03 [0.08], respectively). These findings highlight paternal and maternal preconception RBC folate, and imbalances in OCM metabolites are associated with an increased SPL risk, offering novel insights into SPL etiology and have public health implications.

## Graphical abstract

One‐carbon metabolism (OCM) nutrients play a critical role in fetal development and are implicated in the etiology of spontaneous pregnancy loss (SPL). In our large cohort study, we report for the first time that paternal red blood cell folate levels ≥ 400 ng/mL—consistent with the World Health Organization's recommendation for neural tube defect prevention—are associated with a 64.0% reduction in SPL risk. Targeted metabolomic profiling further identified distinct preconception OCM metabolites in both parents that correlated with SPL risk. We provide novel human evidence underscoring the importance of paternal folate status and OCM homeostasis, alongside maternal factors, in the pathophysiology of SPL, with considerable implications for public health and preconception care.

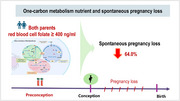

## 1 Introduction

Spontaneous pregnancy loss (SPL) stands as a pivotal reproductive complication, often foreshadowing an elevated risk of infertility and recurrent pregnancy loss [1]. Its global prevalence estimates vary substantially (2.8%–20%) due to heterogeneous diagnostic criteria and methodological differences across studies [2, 3, 4]. While maternal factors—including demographic, genetic, anatomical, infectious, endocrine, and immune dysregulations—contribute to pathogenesis, over 50% of SPL cases remain unexplained [5], underscoring the need for novel etiological insights.

One‐carbon metabolism (OCM) nutrients are indispensable for early embryonic development, providing one‐carbon units for the biosynthesis of DNA, proteins, lipids, and for genome epigenetic regulation [6]. Folic acid and vitamin B12 are among the most well‐known and essential OCM nutrients in preconception care. Folic acid supplementation in women of childbearing age elevates red blood cell (RBC) folate concentrations—the World Health Organization's gold‐standard biomarker for preconception folate status—thereby preventing offspring neural tube defects (NTDs) and congenital heart defects [7, 8]. However, associations of maternal circulating folate levels with SPL risk remain inconsistent [9, 10, 11], with study methodology differed in the timing of measuring folate status or used suboptimal biomarkers. To date, whether maternal preconception RBC folate is associated with the risk of SPL remains uninvestigated. Vitamin B12, another OCM nutrient and a cofactor in both the folate and methionine metabolism cycles, impairs placental development by disrupting folate cycling [12]. High‐quality evidence regarding maternal periconceptional vitamin B12 and its interplay with folate in relation to SPL is lacking [13]. These critical scientific questions call for a well‐designed large cohort study to address them.

Emerging evidence suggests that paternal factors make a significant contribution to offspring health, both through the genetic composition of the fetus and placenta and by influencing OCM nutrient levels [14]. Murine models indicate that paternal folate deficiency increases post‐implantation loss and congenital anomalies [15]. However, human studies are scarce, limited by the challenges of prospectively assessing paternal preconception biomarkers and pregnancy outcomes in large cohorts. Furthermore, OCM nutrients from both parents converge to regulate key processes such as genomic imprinting, placental angiogenesis, and epigenetic programming in the early conceptus. Nevertheless, the interplay between preconception OCM nutrient status in couples and their joint association with SPL risk remains virtually unexplored. Elucidating this dyadic contribution is crucial for moving beyond singular explanations toward a more holistic understanding of SPL etiology.

**FIGURE 1:**
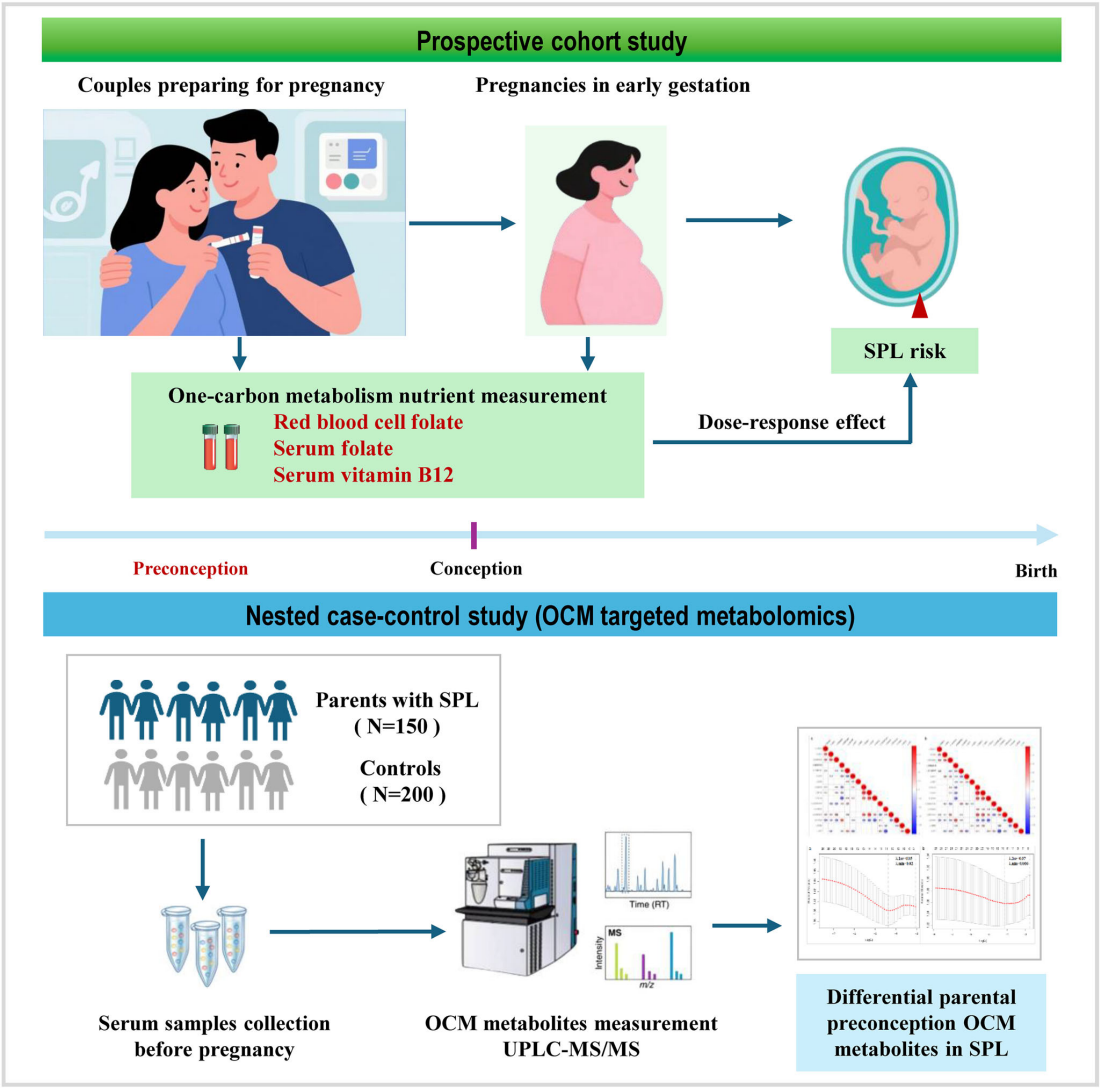
Workflow diagram of this study. OCM, one‐carbon metabolism; SPL, spontaneous pregnancy loss.

We hypothesize that unbalanced maternal and paternal OCM nutrient levels before conception may significantly influence the risk of SPL. Here, we quantified the associations of paternal and maternal preconception folate and vitamin B12 levels with subsequent risk of SPL in a large‐scale prospective cohort, and identified differential preconception OCM metabolites associated with SPL risk in both parents using targeted metabolomics in an exploratory nested case–control subcohort (Figure 1).

## 2 Results

### 2.1 Baseline Characteristics

**FIGURE 2:**
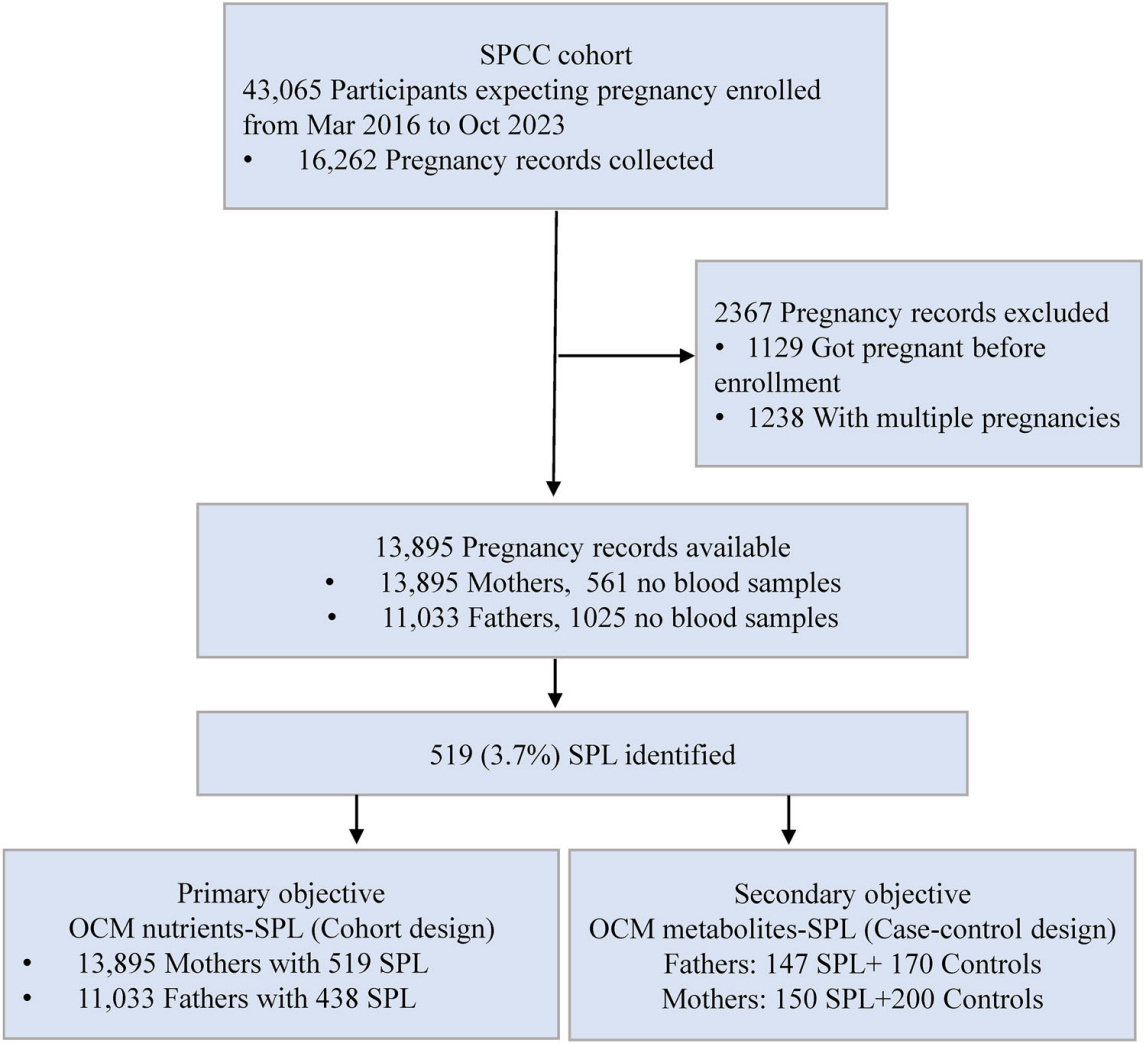
Flow chart of this study. OCM, one‐carbon metabolism; SPCC, Shanghai Preconception Cohort Study; SPL, spontaneous pregnancy loss.

A total of 13,895 eligible pregnancy records were included in the final analysis, with 519 (3.7%) cases of SPL occurring (Figure 2). The fathers and mothers were aged 30.0 (3.9) and 28.9 (3.5) years at enrollment, and 37.1% and 12.5% of them were overweight or obese, respectively (Table 1). Over one‐third of families had an abnormal pregnancy history. Note that 29.1% of fathers were smokers and 60.8% were alcohol users, while the proportions were much lower in mothers (1.6% and 28.8%). Couples who developed SPL were generally older, had higher serum folate levels and a higher prevalence of abnormal pregnancy history, and had lower RBC folate concentrations in fathers only (187.0 [132.2, 240.8] vs. 196.8 [144.0, 270.6] ng/mL; Table S1).

**TABLE 1:** Baseline characteristics of participants.

Variables	Fathers	Mothers	Mothers' subgroup^d^
*N*	11,033	13,895	4203
Age, mean (SD), years	30.0 (3.9)	28.9 (3.5)	27.9 (3.3)
< 35 years	9821 (89.0)	13,066 (94.0)	4067 (96.8)
≥ 35 years	1207 (10.9)	826 (5.9)	136 (3.2)
Missing	5 (< 0.1)	3 (< 0.1)	0
Preconception BMI, mean (SD), kg/m^2^	24.0 (3.3)	21.0 (2.8)	20.8 (2.6)
<24 kg/m^2^ ^a^	4635 (42.0)	12,051 (86.7)	3721 (88.5)
≥24 kg/m^2^	4093 (37.1)	1731 (12.5)	480 (11.4)
Missing	2305 (20.9)	113 (0.8)	2 (< 0.1)
Education			
High school	730 (6.6)	942 (6.8)	257
College	8227 (74.6)	10,403 (74.9)	2984
Postgraduate	2051 (18.6)	2475 (17.8)	950
Missing	25 (0.2)	75 (0.5)	12
Smoking exposure	3207 (29.1)	222 (1.6)	30 (0.7)
Missing	81 (0.7)	91 (0.7)	29 (0.7)
Drinking	6706 (60.8)	3996 (28.8)	1045 (24.8)
Missing	99 (0.9)	132 (0.9)	24 (0.6)
Gravidity			
1	6611(60.0)	8317 (59.9)	2299 (54.7)
> 1	3635 (32.9)	4682 (33.7)	1406 (33.5)
Missing	787 (7.1)	896 (6.4)	498 (11.8)
Abnormal pregnancy history	4522 (41.0)	4662 (33.6)	1378 (32.8)
Missing	55 (0.5)	0	0
RBC folate, median (IQR), ng/mL	196.3 (143.0–269.3)	222.8 (162.5–316.7)	235.7 (170.7–339.3)^e^
< 400 ng/mL^b^	8177 (74.2)	10,630 (76.5)	3347 (79.6)
≥ 400 ng/mL	667 (6.0)	1647 (11.9)	698 (16.6)
Missing	2189 (19.8)	1618 (11.6)	158 (3.8)
Serum folate, median (IQR), nmol/L	6.2 (4.5–8.8)	9.3 (6.5–13.3)	9.1 (6.5–12.9)^e^
Missing	1441 (13.1)	1489 (10.7)	95 (2.3)
Vitamin B12, median (IQR), pg/mL	394.0 (307.0–508.0)	496.0 (379.0–639.0)	485.0 (373.0–619.0)^e^
Deficiency (< 200 pg/mL)^c^	382 (3.5)	292 (2.1)	45 (1.1)
Missing	1467 (13.3)	1194 (8.6)	92 (2.2)
Duration between enrollment and conception, median (IQR), month	12.4 (11.9)	12.6 (12.2)	10.4 (9.0)
Missing	719 (6.5)	1548 (11.1)	55 (1.3)

*Note*: Continuous data were summarized as mean (SD) or median (IQR), and categorical data were displayed as percentages. Unless otherwise indicated, data were expressed as number (%) of subjects.

Abbreviations: BMI, body mass index; IQR, interquartile range; RBC, red blood cell; SD, standard deviation.

^a^ The BMI was categorized according to the definitions for the Chinese population.

^b^ The cut‐off value for RBC folate of 400 ng/mL was chosen following the World Health Organization guidelines for NTDs.

^c^ The 200 pg/mL cut‐off for vitamin B12 deficiency was categorized according to the published review by Oh et al. [16].

^d^ The mothers with OCM nutrient levels at early gestation.

^e^ The OCM nutrient levels measured at early gestation.

### 2.2 Associations of Preconception OCM Nutrient Levels With SPL Risk

**FIGURE 3:**
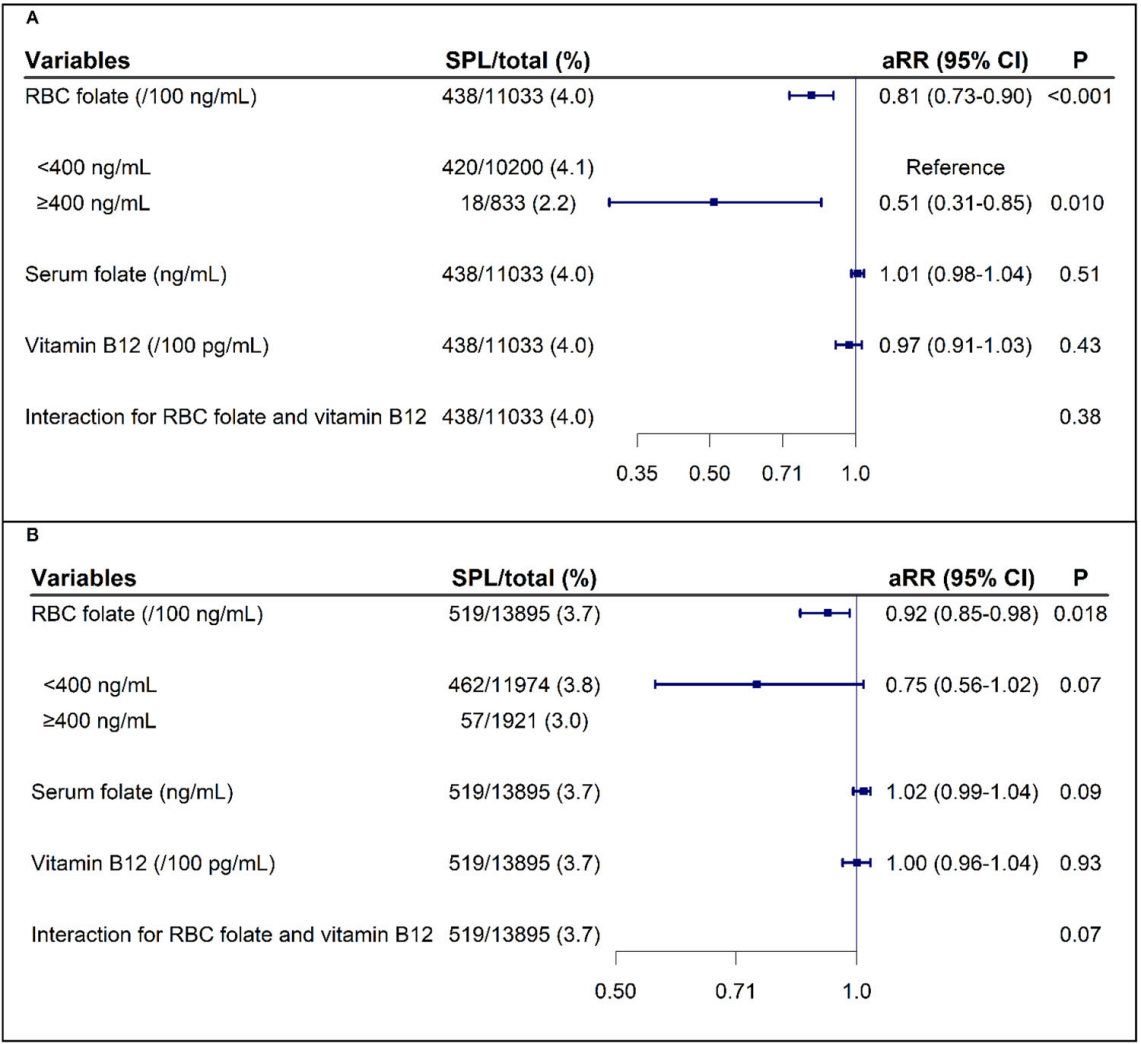
Associations of parental preconception OCM nutrient levels with SPL risk. A, father; B, mother. Paternal OCM nutrient levels were analyzed using 11,033 fathers from couples (A), while maternal levels were analyzed using all 13,895 mothers, including those from couples and single mothers (B). For the interaction analysis, RBC folate was categorized as a binary variable (< 400 vs. ≥ 400 ng/mL), and vitamin B12 was treated as a continuous variable. The axis was log‐scaled. RBC, red blood cell; RR, risk ratio; SPL, spontaneous pregnancy loss.

As shown in Figure 3, each 100 ng/mL increase in paternal RBC folate concentrations was significantly associated with a lower SPL risk (adjusted risk ratio [aRR], 0.81; 95% confidence interval [CI], 0.73–0.90), and RBC folate ≥ 400 ng/mL was associated with a 49% reduced risk of SPL (aRR, 0.51; 95% CI, 0.31–0.85). A similar, though weaker, association was observed for continuous maternal RBC folate (aRR, 0.92; 95% CI, 0.85–0.98) and for the dichotomized status (aRR, 0.75; 95% CI, 0.56–1.02). No significant associations were found for serum folate or vitamin B12 levels. Based on the observed associations between parental preconception RBC folate concentrations and SPL, the *E*‐values (CI) were 1.77 (1.39) and 1.39 (1.16) for the association of paternal and maternal RBC folate, respectively (Figure S1).

**FIGURE 4:**
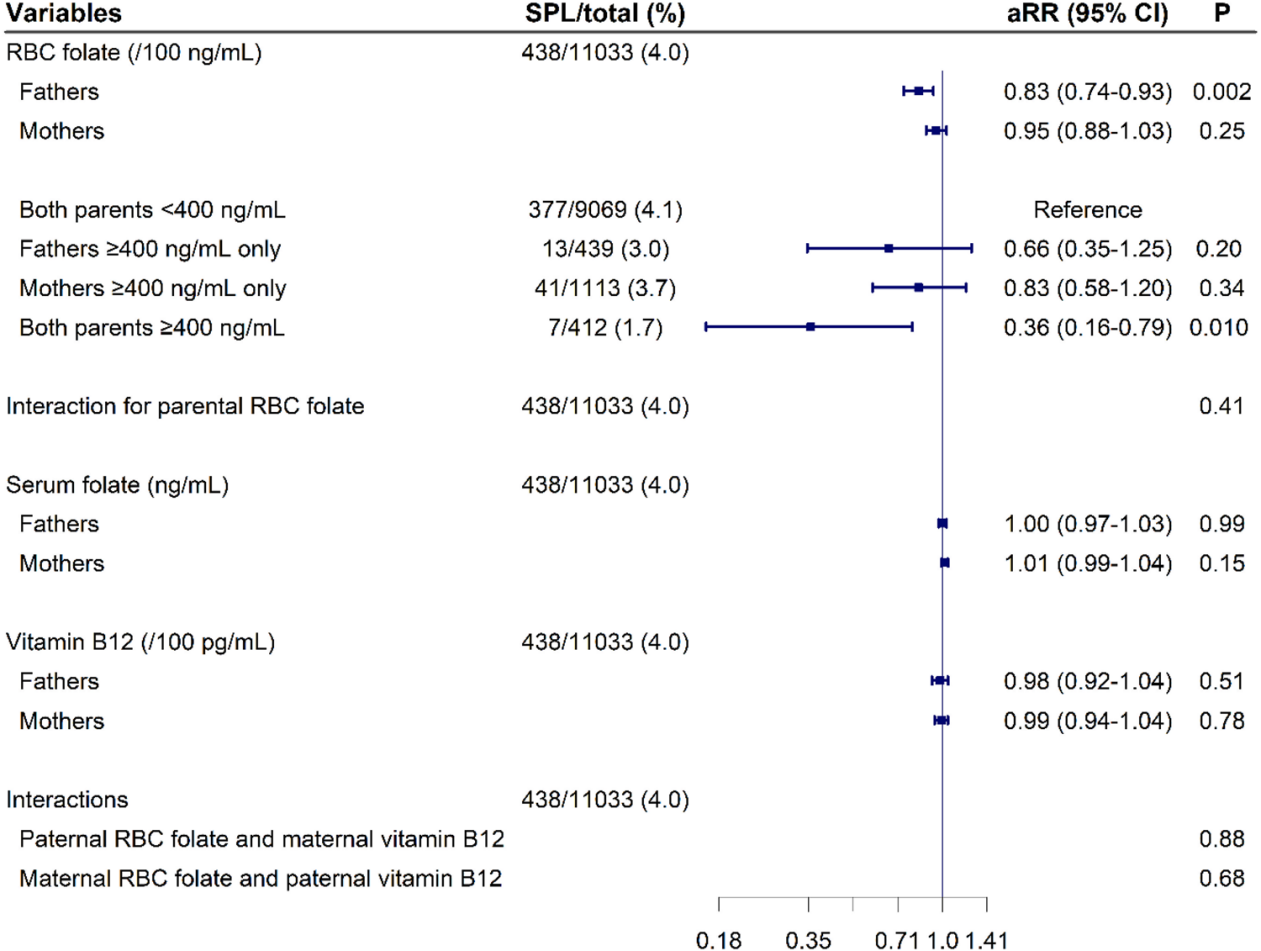
Comparison of parental preconception OCM nutrient levels with SPL risk in 11,033 complete couples. For the interaction analysis, RBC folate was categorized as a binary variable (< 400 vs. ≥ 400 ng/mL), and vitamin B12 was treated as a continuous variable. The axis was log‐scaled. RBC, red blood cell; SPL, spontaneous pregnancy loss.

In the combined analysis (Figure 4), the significant associations of paternal RBC folate with SPL remained and were independent of maternal levels (aRR, 0.83; 95% CI, 0.74–0.93), whereas the association of maternal RBC folate became weaker and nonsignificant. Further combined analyses based on dichotomized folate showed some evidence of a reduced risk of SPL when either paternal or maternal RBC folate was ≥ 400 ng/mL (with reductions of 34% and 17%, respectively) compared with both parental RBC folate < 400 ng/mL. However, the reduction of SPL risk increased to 64.0% and reached statistical significance only when both parents RBC folate ≥ 400 ng/mL (aRR, 0.36; 95% CI, 0.16–0.79). Trend analyses based on ordinal paternal and maternal preconception RBC folate concentrations showed significant results (*p* values were < 0.001 and 0.027, respectively; Figure S2). Restricted cubic spline (RCS) models suggested linear relationships of continuous parental preconception RBC folate concentrations with SPL (*p* for nonlinearity = 0.08 for fathers, 0.68 for mothers; Figure S3). Sensitivity analyses using different SPL definitions, varying exclusion criteria, and addressing potential outcome under‐ascertainment and adjustments for parental educational level all yielded results consistent with the primary findings (Figures S4–S9). The subgroup analyses showed that the inverse associations between paternal RBC folate and SPL were likely stronger in the ≥35‐year group (aRR, 0.68; 95% CI, 0.52–0.89) than in the <35‐year group (aRR, 0.84; 95% CI, 0.75–0.95; Figure S10), while no significant interactions were observed between parental RBC folate and subgroups. Results were consistent with the main analyses when stratified by preconception BMI, smoking exposure, and alcohol consumption.

In the sub‐cohort of 4203 pregnancies with maternal blood samples collected during early gestation and 121 SPL cases (Table S2), no associations were observed for continuous or dichotomized maternal RBC folate at early gestation (aRRs [95% CIs]: 1.04 [0.96–1.14] and 1.10 [0.76–1.58], respectively) or for serum folate or vitamin B12 levels (1.01 [0.96–1.06] and 1.09 [0.99–1.19], respectively; Figure S11).

### 2.3 Associations of Parental Preconception OCM Metabolomics With SPL

**FIGURE 5:**
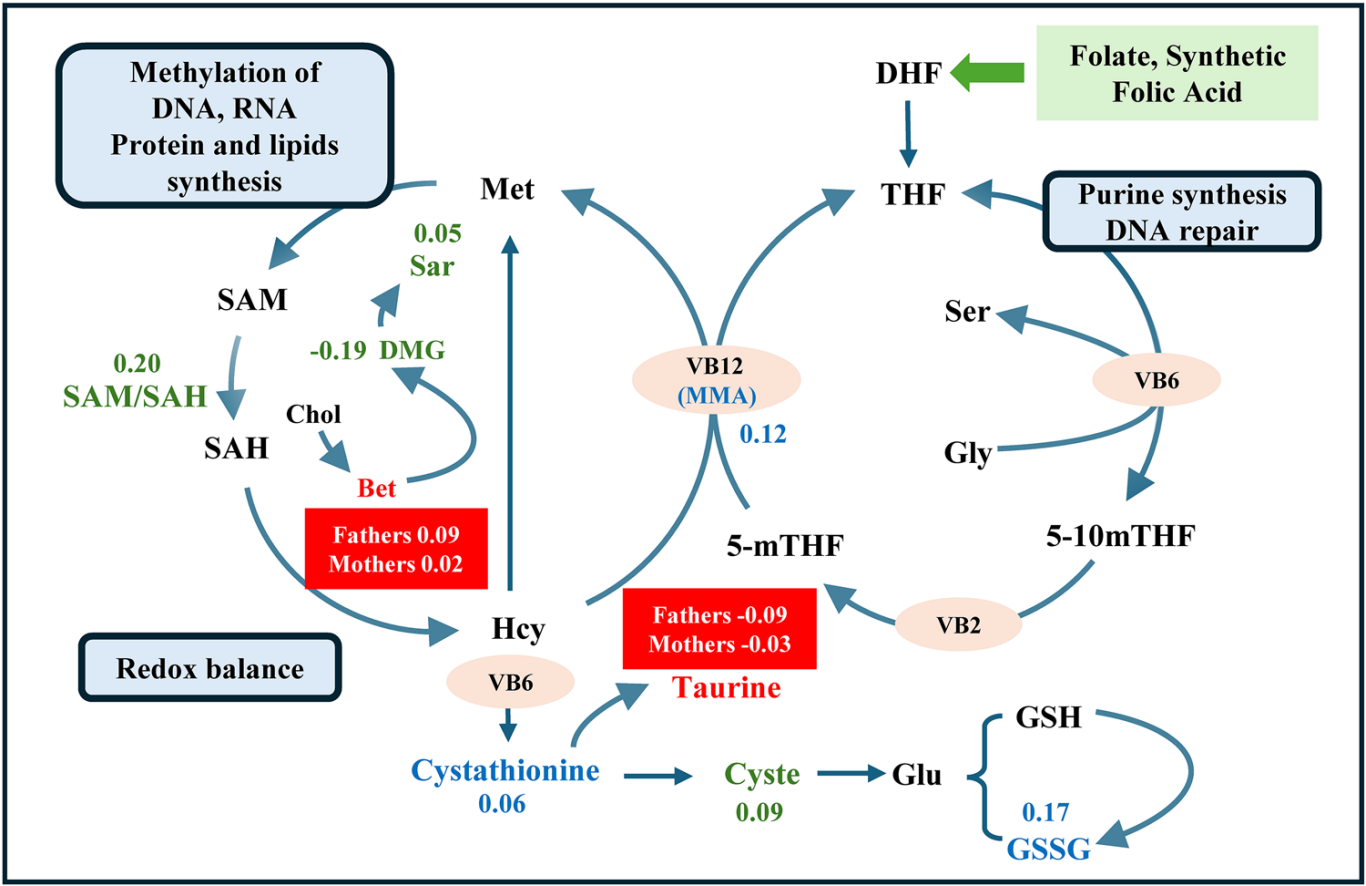
Diagram of the relationship between parental preconception one‐carbon metabolism metabolites and SPL. Substrates in blue and green represent the father's and mother's β coefficients from the LASSO regression model, respectively, while those in red denote combined parental β coefficients. Age, preconception BMI, smoking, and drinking status were treated as covariates, and preconception RBC folate and OCM metabolites were treated as exposures in the LASSO regression model. Metabolites normalized into *z*‐scores prior to analysis. Bet, betaine; Chol, choline; Cyste, Cysteine; DHF, dihydrofolate; Gly, Glycine; GSSG, oxidized glutathione; GSH, glutathione; Hcy, homocysteine; Met, methionine; MMA, methylmalonic acid; SAM, S‐adenosylmethionine; SAH, S‐adenosylhomocysteine; Sar, Sarcosine; Ser, serine; THF, tetrahydrofolate; 5‐mTHF, 5‐methyltetrahydrofolate.

The demographic characteristics of participants in this case–control sample were similar to those of the overall SPCC cohort (Figure S12, Table S3), and 16 metabolites were moderately to strongly cross‐correlated with each other (Figure S13). The exploratory case–control analysis identified differential associations of OCM metabolites with SPL risk in parents. Paternal GSSG, cystathionine, and MMA levels (β coefficients [bootstrap SE]: 0.17 [0.11], 0.09 [0.11], and 0.12 [0.10], respectively; Table S4 and Figure 5) and maternal sarcosine, SAM/SAH ratio, and cysteine levels (0.05 [0.11], 0.20 [0.11], and 0.09 [0.10], respectively) were associated with SPL risk, while an inverse association was observed for maternal DMG (−0.19 [0.15]). Betaine levels in both parents were associated with SPL risk (0.09 [0.11] for fathers and 0.02 [0.08] for mothers, respectively), whereas taurine demonstrated inverse associations (−0.09 [0.11] for fathers and −0.03 [0.08] for mothers, respectively).

## 3 Discussion

In this large prospective cohort study, higher preconception RBC folate concentrations in both fathers and mothers were independently associated with a lower SPL risk in a dose–response manner. Specifically, each 100 ng/mL increase in RBC folate was associated with a 19% reduction in SPL risk for fathers and an 8% reduction for mothers. Achieving preconception RBC folate concentrations of 400 ng/mL in both parents, fathers only or mothers only were associated with a 64%, 34%, and 17% reduced risk of SPL, respectively. Notably, these associations were not observed for early‐gestation RBC folate or for serum folate or vitamin B12 before or after conception. Targeted metabolomics analysis identified distinct parental preconception OCM metabolites associated with SPL risk, implicating the role of OCM homeostasis in SPL pathogenesis and conveying substantial clinical and public health relevance. To our knowledge, this is the first study to investigate parental preconception RBC folate simultaneously with subsequent SPL risk on such a scale. Our findings, particularly regarding paternal folate, contribute to elucidating the pathology of SPL.

While fathers contribute half of the genetic material to the fetus and the placenta, the impact of paternal preconception folate on reproductive outcomes has remained largely underexplored. Our study is the first to quantify this relationship in a prospective cohort, identifying a dose–response association between paternal RBC folate and SPL risk that remains independent of maternal folate levels and other extensive covariates. Our results contrast with those from previous placebo‐controlled randomized trials involving infertile men, which found no significant effect of high‐dose folic acid on SPL incidence [17]. This discrepancy arises in part likely from differences in population characteristics and the biomarkers used. Unlike prior studies that relied on serum folate, we utilized RBC folate, a more reliable marker of tissue folate levels [18], thereby enhancing the validity of our findings. Moreover, relatively low baseline folate levels in this population may amplify the observed associations [19]. Interestingly, the magnitude of the association appeared stronger in men aged ≥ 35 years compared to younger participants (RR = 0.68 vs. RR = 0.84), suggesting greater attention for improving RBC folate concentrations in fathers with advanced age.

Our findings provide novel population‐based evidence linking maternal preconception RBC folate concentrations to SPL risk, aligning with well‐established associations between maternal folate status and reduced risks of NTDs and congenital heart defects [18, 20]. This study extends the evidence base to include SPL, emphasizing the critical role of maternal folate in early embryonic development. Mechanistically, insufficient maternal folate impairs DNA synthesis, methylation, and placental function, increasing the risk of embryonic loss [6]. To date, only one small case–control study in Spain reported an association of maternal RBC folate levels below 400 ng/mL (906 nmol/L) with a doubled risk of early gestational SPL [11]. However, that study was limited by its retrospective design, as blood samples were collected 1 week after the diagnosis of pregnancy loss, complicating causal inference. Notably, the absence of an association for early‐gestation RBC folate in our prospective cohort further highlights the preconception period as the critical window for folate optimization. This explanation aligns with a previous study that interventions during preconception, rather than gestation, yield the most substantial benefits for pregnancy outcomes [21].

Emerging evidence supports the biological plausibility of paternal folate's role in SPL etiology. Previous animal and human studies indicate that paternal folate deficiency impairs sperm quality, disrupts placental development [22], and increases embryonic loss [14], likely through mechanisms such as impaired sperm DNA integrity and altered epigenetic programming. These paternal factors may also influence maternal immune tolerance and the uterine environment [23], while affecting placental folate transport—a process critical for embryonic development [24]. Our observation of a stronger association for paternal RBC folate than for maternal levels may reflect lower folate levels in fathers or unique paternal contributions to early development. Our metabolomics findings provide mechanistic support: elevated paternal GSSG (a marker of oxidative stress) and altered betaine levels suggest that folate deficiency may exacerbate oxidative damage to paternal DNA or disrupt methyl donor availability. Concurrently, maternal imbalances in the SAM/SAH ratio (a key index of methylation potential) and cysteine levels (linked to redox homeostasis) point toward impaired embryonic methylation dynamics and trophoblast function [25, 26]. Notably, the consistent associations of betaine and taurine in both parents with SPL suggest that shared underlying mechanisms, specifically impaired methylation regulation and redox imbalance, underlie the role of parental OCM homeostasis in SPL pathogenesis.

Our study highlights the need to re‐evaluate the current recommendations for folic acid supplementation. While the WHO establishes a 400 ng/mL RBC folate target for women primarily to prevent NTDs, our findings provide novel evidence that reaching this threshold before conception offers additional benefit in preventing SPL. Notably, paternal preconception RBC folate reaching at least 400 ng/mL may be effective for early prevention of SPL. A prior prospective study based on an assisted reproduction cohort found no additional benefits to embryonic growth when paternal RBC folate exceeded 528 ng/mL [14]. While not directly generalizable to SPL, we have reasons to suggest that an upper limit level of RBC folate of 500 ng/mL may be appropriate and safe for fathers. Reaching the RBC folate target of 400–500 ng/mL, particularly in regions without mandatory folic acid fortification, requires integrated public health strategies including dietary interventions and supplementation programs for both parents. Additionally, the absence of significant associations for serum folate in our cohort underscores the necessity of using RBC folate as a reliable measurement of long‐term folate status [27]. Although vitamin B12 is crucial for OCM, its role in SPL etiology remains unclear and warrants further investigation.

These findings carry significant public health implications. Strategies to improve parental RBC folate status before conception, particularly in regions without mandatory folic acid fortification, may reduce population SPL risk. Further research to establish evidence‐based guidelines for optimizing paternal folate status is needed. We provide new evidence proposing the necessity of assessing OCM nutritional balance and functional biomarkers in both parents before conception, moving beyond the current focus on isolated nutrients or maternal factors alone. Personalized interventions may become possible by identifying high‐risk groups for SPL risk via comprehensively assessing related key OCM metabolites, dietary intake, and key genetic variants. Implementing public health guidelines that promote couple‐based approaches to OCM modulation, for example, by incorporating folate assessment into preconception care as the first step, would represent an essential step forward.

To our knowledge, our study is the first and largest population‐based prospective cohort study that simultaneously evaluates the impact of parental OCM nutrients on SPL risk. Nevertheless, this study has limitations. First, as an observational study, residual confounding cannot be completely ruled out; for instance, genetic variants in key OCM enzymes were not adjusted for. However, our *E*‐value analysis confirms the robustness of our findings: an unmeasured confounder would need an RR of at least 1.77 (for fathers) or 1.39 (for mothers) with both RBC folate and SPL to nullify the observed associations. Certain factors, such as chromosomal abnormalities and severe malformations [4], showed a strong association with SPL but contributed little to the overall high prevalence of SPL due to their rarity. Second, the generalizability of our results may be limited, as our participants were from Eastern China—a region with relatively low folate levels compared to countries with mandatory fortification. Third, our metabolomics analysis was based on small sample size and without multiple testing adjustment so that the findings were for hypothesis‐generating only. Future validation in larger, more diverse populations is essential before definitive causal inferences can be made.

## 4 Conclusion

Our study provides robust evidence that higher paternal and maternal preconception RBC folate concentrations were independently associated with reduced risk of SPL, with a particularly pronounced effect observed for paternal folate status. Reaching the WHO‐recommended threshold of 400 ng/mL in both parents before conception was associated with a 64% reduction in SPL risk, a finding that underscores the critical yet often overlooked role of paternal preconception health. These results suggest that OCM homeostasis in both partners is a key determinant of pregnancy maintenance. Public health strategies should move toward a couple‐based paradigm in preconception care, where monitoring and optimizing RBC folate levels in both parents may serve as an effective strategy for reducing the global burden of SPL. Future interventional trials are warranted to confirm the efficacy of dual‐parent folate optimization in improving pregnancy outcomes.

## 5 Methods

### 5.1 Study Design and Population

This study's population was drawn from the ongoing Shanghai Preconception Cohort (SPCC, NCT02737644) [28]. Couples planning for pregnancy and aged 18–45 years who enrolled between March 2016 and October 2023 and had a clear record of pregnancy outcome by October 2023 were eligible. Participants who were found to be pregnant at study enrollment were excluded. We retained their first pregnancy outcome for analysis for the families with a second or third pregnancy in the cohort. The study population comprised 11,033 complete couples and 2862 unpaired mothers whose partners were not enrolled (e.g., due to absence at the time of recruitment). Parental OCM nutrient levels were analyzed using the maximum number of fathers (*n* = 11,033) and mothers (*n* = 13,895), and the combined parental analyses were restricted to the 11,033 complete couples.

For targeted metabolomics profiling, we randomly selected 150 SPL cases (with 150 mothers and 147 fathers) and 200 controls (with 200 mothers and 173 fathers) for measurement of OCM metabolites. Additionally, blood samples at early gestation (< 13 weeks of gestation) were successfully collected from a subgroup of mothers. Details of the sample selection and the assay are described in the Supporting Information. The study adhered to the Strengthening the Reporting of Observational Studies in Epidemiology (STROBE) reporting guideline.

### 5.2 Outcome

We defined the SPL cases as fetal death occurring at any stage of gestation, with corresponding codes from the International Statistical Classification of Diseases, Tenth Revision (Table S5). We abstracted and confirmed SPL cases through the hospital‐ and city‐level electronic medical records systems. For couples who migrated to a different city after enrolment, we confirmed the diagnosis via telephone interviews.

### 5.3 Measurement of OCM Nutrients and Covariates

Venous blood samples were collected from the participants at enrollment according to standard operating procedures [28]. We utilized light‐proof tubes and procedures during isolation, transportation, and storage. RBC folate, serum folate, and vitamin B12 levels were measured using electrochemiluminescence assays (ARCHITECT i2000SR Analyzer; Abbott Laboratories, USA) in the central laboratory of the Children's Hospital of Fudan University. Three standard solutions (quality controls [QC]1, QC2, and QC3) were used as daily QCs. The inter‐assay coefficients of variation of QC1–QC3 for RBC folate, serum folate, and vitamin B12 were < 7.5%, and the intra‐assay coefficients of variation were < 6.5% for the entire study population. Targeted metabolomics (Shanghai Metabolome Institute) using UPLC‐MS/MS (Agilent, USA) was employed to measure 16 serum OCM metabolites categorized into amino acids (glycine, L‐serine, cystathionine, taurine, L‐methionine, L‐cysteine, oxidized L‐glutathione [GSSG]), choline metabolites (choline, betaine, dimethylglycine [DMG], and sarcosine), folate metabolites (total homocysteine [tHcy], S‐adenosylmethionine [SAM], and S‐adenosyl homocysteine [SAH]), and water‐soluble vitamins (5‐methyltetrahy drofolate [5‐mTHF] and methylmalonic acid [MMA]).

**FIGURE 6:**
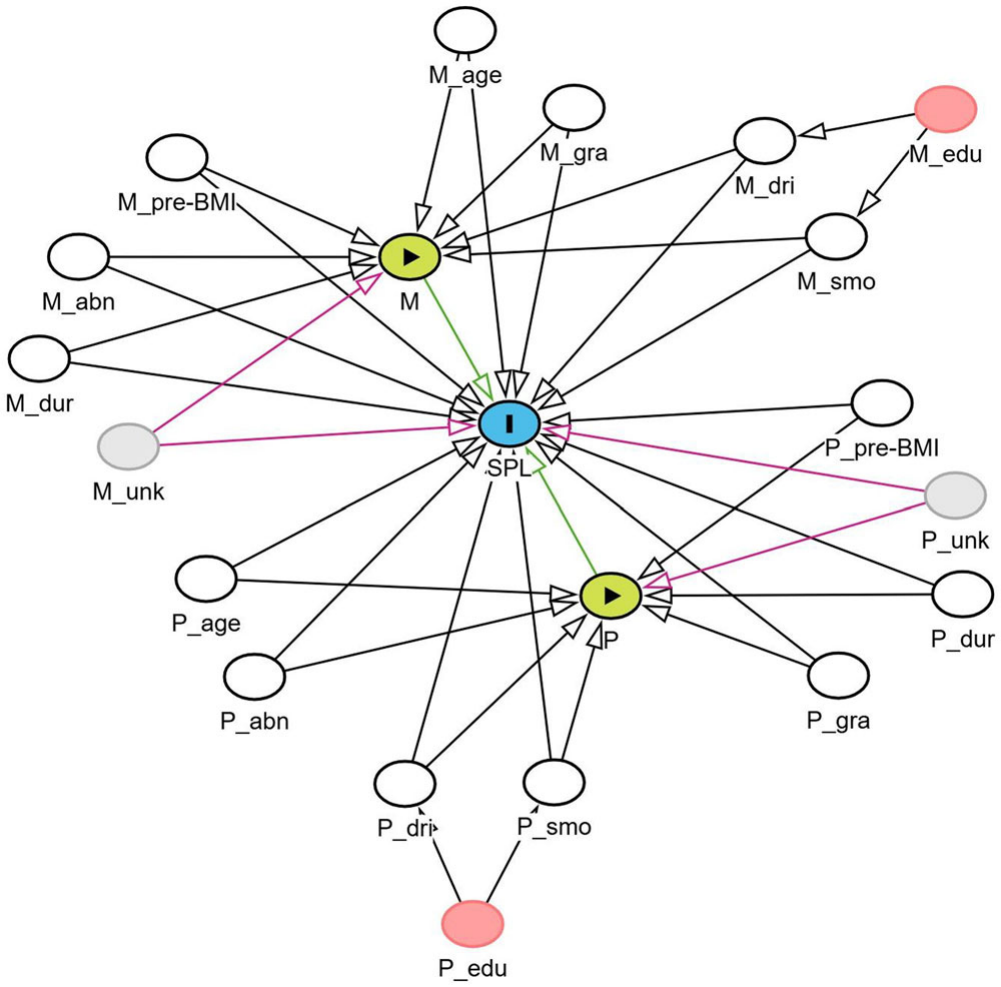
Directed acyclic graph documenting assumptions about the association between covariates, exposure, and outcome. M and P represent the maternal and paternal one carbon metabolism nutrient levels before pregnancy (exposures). SPL, spontaneous pregnancy loss (outcome). The white circles represent the adjusted covariates, and the pink circles represent the unadjusted variables. We assumed that educational level influences smoking and alcohol drinking; therefore, we adjusted for smoking and alcohol drinking instead of educational level in the regression model. M_age, maternal age; M_abn, maternal abnormal pregnancy history; M_dur, maternal duration between enrollment and conception; M_edu, maternal education level; M_dri, maternal alcohol drinking status; M_gra, maternal gravidity; M_pre‐BMI, maternal preconception BMI; M_smo, maternal smoking exposures; M_unk, maternal unmeasured variables; P_age, paternal age; P_abn, paternal abnormal pregnancy history; P_dur, paternal duration between enrollment and conception; P_edu, paternal education level; P_dri, paternal alcohol drinking status; P_gra, paternal gravidity; P_pre‐BMI, paternal preconception BMI; P_smo, paternal smoking exposures; P_unk, paternal unmeasured variables.

As described elsewhere, parental demographics and lifestyle information were obtained at enrollment through a standardized self‐administered questionnaire [28]. We utilized a Directed Acyclic Graph (DAG) to guide the selection of covariates in the following association analysis (Figure 6) [29]. Pre‐pregnancy BMI was calculated through body weight and height and categorized as normal weight (< 24 kg/m^2^) or overweight (≥ 24 kg/m^2^) [30]. Smoking exposure within 3 months before or during pregnancy was defined as self‐smoker or exposed to second‐hand cigarette smoking (binary variable, yes vs. no), and alcohol drinking was defined as consuming any alcoholic beverages (binary variable, yes vs. no) during the same period. We defined a history of adverse pregnancy outcomes as the occurrence of one or more prior events, including abortion, preterm delivery, stillbirth, or ectopic pregnancy. The duration between enrollment and conception was calculated by subtracting the last menstrual date from the recruitment date.

### 5.4 Statistical Analysis

Our primary aim was to quantify the associations of parental preconception RBC folate, serum folate, and vitamin B12 concentrations with subsequent SPL risk. We used generalized linear models (GLM) with binomial family and log link function fitted by the iterated reweighted least squares method to estimate the RRs and 95% CIs (Figure S14). In the primary analyses, we treated paternal and maternal nutrient concentrations as continuous variables in separate models and subsequently in a combined model, all adjusted for corresponding to the same confounders. Then, we repeated the modeling approach using dichotomized nutrient concentration variables while adjusting for the identical confounders. We conducted the following sensitivity analyses to test the robustness of the primary findings. First, we repeated the primary analysis models applying three alternative SPL definitions [31, 32, 33] (Table S6). Second, we additionally adjusted for education level, a socioeconomic confounder that was presented in the DAG and may affect both nutrient status and pregnancy outcomes. Third, we repeated the covariate‐adjusted models after excluding fathers and mothers with missing blood samples to avoid bias from missing data (Figure 2). Moreover, to address potential under‐ascertainment of the outcome, especially of very early SPL cases that might not have led to a hospital visit, we repeated the primary analysis models in a sub‐cohort of 4921 couples (enrolled after November 1, 2018). We also assessed nonlinear relationships of continuous nutrient levels with SPL using an RCS regression model with 3 knots. We further validated the primary analyses by examining maternal continuous nutrient levels during early gestation in a sub‐cohort. For the secondary aim, we applied logistic LASSO regression with SPL as the dependent variable and all measured metabolites as independent variables, allowing us to reduce overfitting, improve model generalizability, and enhance interpretability. To assess the potential impact of unmeasured confounders on the observed associations of main results, we performed an *E*‐value analysis [34]. Statistical analyses were performed using StataSE version 16.0 (Stata Corp LLC, College Station, TX, USA) and R 4.3.1, and all statistical tests were two‐sided at a significance level of 0.05. Detailed statistical methods were provided in the Supporting Information.

## Author Contributions

Study concept and design: Weili Yan, Guoying Huang, and Xiaotian Chen. Methodology: Yi Zhang, Qing Yang, Hong Zhu, Jianwei Hu, Jian Huang, Longmei Jin, Xiaohua Zhang, Yalan Dou, Wennan He, Yuanchen He, Hongyan Chen, Qinyu Yao, and Yuanzhou Peng. Acquisition, analysis, or interpretation of data: Xiaotian Chen, Yi Zhang, and Qinyu Yao. Writing – original draft: Xiaotian Chen. Writing – review and editing: Weili Yan, Guoying Huang, and Xiaotian Chen. Validation: Weili Yan, Xiaojing Ma, and Wei Sheng. Supervision: Weili Yan and Guoying Huang. Funding acquisition: Weili Yan, Guoying Huang, and Xiaotian Chen. All authors have read and approved the final manuscript.

## Funding

This work was supported by the National Key Research and Development Program of China (grant number 2024YFC2707602 and 2021YFC2701004), National Natural Science Foundation of China (grant numbers 82070323, 82373584, and 82204051), Shanghai Sailing Program (grant number 22YF1403900), Youth Project of Shanghai Municipal Commission of Health (grant number 20214Y0439), and the Chinese Academy of Medical Sciences Innovation Fund for Medical Sciences (grant number 2019‐I2M‐5‐002).

## Ethic Statement

The study protocol was approved by the Ethics Committee of the Children's Hospital of Fudan University, Shanghai, China (Approval Number: 2018–151; NCT02737644). Written informed consent was obtained from all participants before recruitment.

## Conflicts of Interest

The authors declare no conflicts of interest.

## Supporting information




**Supporting Figure 1**: E‐value analysis for the observed association. **Supporting Figure 2**: Dose‐response relationship between parental RBC folate levels before pregnancy with SPL risk. **Supporting Figure 3**: Restricted cubic spline plots for the association between parental preconception OCM nutrient levels and SPL risk. **Supporting Figure 4**: Sensitivity analysis by defining SPL according to the Chinese guidelines. **Supporting Figure 5**: Sensitivity analysis by defining SPL according to the European Society of Human Reproduction and Embryology guidelines. **Supporting Figure 6**: Sensitivity analysis by defining SPL according to the WHO guidelines. **Supporting Figure 7**: Sensitivity analysis by excluding the 561 female and 1025 male participants with no blood samples. **Supporting Figure 8**: Sensitivity analysis by addressing potential under‑ascertainment of SPL. **Supporting Figure 9**: Sensitivity analysis by adjusting covariates including parental education level. **Supporting Figure 10**: Subgroup analysis by age group (<35 years, ≥35 years), BMI (<24 years, ≥35 years), smoking exposures (Yes, No) and alcohol drinking (Yes, No). **Supporting Figure 11**: Associations of maternal OCM‐related nutrient levels at early gestation with SPL risk. **Supporting Figure 12**: The distribution of serum OCM metabolites between preconception fathers and mothers. **Supporting Figure 13**: Correlation heatmap for preconception parental OCM metabolites. **Supporting Figure 14**: The statistical analysis plan for this study. **Supporting Figure 15**: Cross validation plot for the penalty term. **Supporting Table 1**: Baseline characteristics of SPL and non‐SPL population. **Supporting Table 2**: Characteristics of the mothers with OCM nutrient levels at early gestation between SPL and non‐SPL (N = 4203). **Supporting Table 3**: Baseline characteristics of participants in the OCM targeted metabolomics study stratified by SPL cases and controls. **Supporting Table 4**: The estimated coefficients for LASSO regression between parental preconception RBC folate, OCM metabolites and SPL risk. **Supporting Table 5**: The ICD‐10 codes used to identify SPL. **Supporting Table 6**: The various definitions of SPL among countries and international organizations. **Other supporting information**: statistical analysis plan, OCM‐targeted metabolomics in SPL, and the Shanghai PreConception Cohort (SPCC) group.

## Data Availability

Data from the SPCC cohort can be shared after reasonable request to the corresponding author.
