# Supplementary material for: Preconception One‐Carbon Metabolism Nutrient Levels in Preparing for Pregnancy Couples and Spontaneous Pregnancy Loss: A Prospective Cohort Study
Source: MedComm (2020). 2026 Mar 30;7(4):e70711. doi: 10.1002/mco2.70711 (PMC13042509; doi:10.1002/mco2.70711)
Supplement: Supplementary file 1 — Supporting Figure 1: E‐value analysis for the observed association. Supporting Figure 2: Dose‐response relationship between parental RBC folate levels before pregnancy with SPL risk. Supporting Figure 3: Restricted cubic spline plots for the association between parental preconception OCM nutrient levels and SPL risk. Supporting Figure 4: Sensitivity analysis by defining SPL according to the Chinese guidelines. Supporting Figure 5: Sensitivity analysis by defining SPL according to the European Society of Human Reproduction and Embryology guidelines. Supporting Figure 6: Sensitivity analysis by defining SPL according to the WHO guidelines. Supporting Figure 7: Sensitivity analysis by excluding the 561 female and 1025 male participants with no blood samples. Supporting Figure 8: Sensitivity analysis by addressing potential under‑ascertainment of SPL. Supporting Figure 9: Sensitivity analysis by adjusting covariates including parental education level. Supporting Figure 10: Subgroup analysis by age group (<35 years, ≥35 years), BMI (<24 years, ≥35 years), smoking exposures (Yes, No) and alcohol drinking (Yes, No). Supporting Figure 11: Associations of maternal OCM‐related nutrient levels at early gestation with SPL risk. Supporting Figure 12: The distribution of serum OCM metabolites between preconception fathers and mothers. Supporting Figure 13: Correlation heatmap for preconception parental OCM metabolites. Supporting Figure 14: The statistical analysis plan for this study. Supporting Figure 15: Cross validation plot for the penalty term. Supporting Table 1: Baseline characteristics of SPL and non‐SPL population. Supporting Table 2: Characteristics of the mothers with OCM nutrient levels at early gestation between SPL and non‐SPL (N = 4203). Supporting Table 3: Baseline characteristics of participants in the OCM targeted metabolomics study stratified by SPL cases and controls. Supporting Table 4: The estimated coefficients for LASSO regression between parental preconc [file MCO2-7-e70711-s001.docx]

**Supplementary appendix**

**Title page**

**Preconception One-Carbon Metabolism Nutrient Levels in Preparing for Pregnancy Couples and Spontaneous Pregnancy Loss: A Prospective Cohort Study**

Xiaotian Chen, ^1^ Yi Zhang, ^2^ Qing Yang,^2^ Hong Zhu,^2^ Jianwei Hu, ^3^ Jian Huang, ^3^ Longmei Jin, ^4^ Xiaohua Zhang, ^4^ Yalan Dou, ^1^ Wennan He,^1^ Yuanchen He, ^1^ Hongyan Chen, ^5^ Qinyu Yao, ^1,2,6^ Yuanzhou Peng, ^1^ Xiaojing Ma, ^2,6^ Wei Sheng, ^2^ Guoying Huang^*^, ^2,6,7^ Weili Yan^*^, ^1,7^ On behalf of the SPCC group

**Running title: One-Carbon Metabolism and Pregnancy Loss**

**Affiliations:**

^1^Department of Clinical Epidemiology & Clinical Trial Unit, Children’s Hospital of Fudan University, National Children’s Medical Center, Shanghai, 201102, China.

^2^The Maternal and Child Healthcare Institute of Songjiang District, Shanghai, 201600, China.

^3^The Maternal and Child Healthcare Institute of Kunshan City, Kunshan, Jiangsu, 215300, China.

^4^Minhang Maternal and Child Health Hospital, Shanghai, 201102, China.

^5^Shanghai Key Laboratory of Birth Defects, Children’s Hospital of Fudan University, National Children’s Medical Center, Shanghai, 201102, China.

^6^Pediatric Heart Center, Children’s Hospital of Fudan University, National Children’s Medical Center, Shanghai, 201102, China.

^7^Research Unit of Early Intervention of Genetically Related Childhood Cardiovascular Diseases (2018RU002), Chinese Academy of Medical Sciences, Shanghai, 201102, China.

**Corresponding authors:**

**Guoying Huang, MD, gyhuang@shmu.edu.cn**

Pediatric Heart Center, Children’s Hospital of Fudan University, National Children’s Medical Center, Shanghai, China & Shanghai Key Laboratory of Birth Defects, Shanghai, China

Address: 399 Wan Yuan Road, Shanghai 201102, People’s Republic of China

Tel: 86-21-64931928

Fax: 86-21-64931002

**Weili Yan, PhD, yanwl@fudan.edu.cn**

Department of Clinical Epidemiology & Clinical Trial Unit, Children’s Hospital of Fudan University, National Children’s Medical Center, Shanghai, China & Shanghai Key Laboratory of Birth Defects, Shanghai, China

Address: 399 Wan Yuan Road, Shanghai 201102, People’s Republic of China

Tel: 86-21-64931215

Fax: 86-21-64931215

**Contents**

**Figure S1.** E-value analysis for the observed association...........................................................................3

**Figure S2**. Dose-response relationship between parental RBC folate levels before pregnancy with SPL risk .............................................................................................................................................................4

**Figure S3.** Restricted cubic spline plots for the association between parental preconception OCM nutrient levels and SPL risk........................................................................................................................5

**Figure S4.** Sensitivity analysis by defining SPL according to the Chinese guidelines…………….........6

**Figure S5.** Sensitivity analysis by defining SPL according to the European Society of Human Reproduction and Embryology guidelines.................................................................................................7

**Figure S6**. Sensitivity analysis by defining SPL according to the WHO guidelines……………….…....8

**Figure S7**. Sensitivity analysis by excluding the 561 female and 1025 male participants with no blood samples.......................................................................................................................................................9

**Figure S8.** Sensitivity analysis by addressing potential under‑ascertainment of SPL…………..............10

**Figure S9.** Sensitivity analysis by adjusting covariates including parental education level……………11

**Figure S10.** Subgroup analysis by age group (<35 years, ≥35 years), BMI (<24 years, ≥35 years), smoking exposures (Yes, No) and alcohol drinking (Yes, No)………………………………………....12

**Figure S11.** Associations of maternal OCM-related nutrient levels at early gestation with SPL risk………………………………………………………………………………………………………13

**Figure S12.** The distribution of serum OCM metabolites between preconception fathers and mothers……………………………………………………………………………………………...…..14

**Figure S13.** Correlation heatmap for preconception parental OCM metabolites……………………....15

**Figure S14.** Statistical analysis plan………………….............................................................................16

**Figure S15.** Cross validation plot for the penalty term………………………………………….……...23

**Table S1**. Baseline characteristics of SPL and non-SPL population........................................................17

**Table S2**. Characteristics of the mothers with OCM nutrient levels at early gestation between SPL and non-SPL (N=4203) ..................................................................................................................................19

**Table S3.** Baseline characteristics of participants in the OCM targeted metabolomics study stratified by SPL cases and controls.............................................................................................................................20

**Table S4.** The estimated coefficients for LASSO regression between parental preconception RBC folate, OCM metabolites and SPL risk................................................................................................................20

**Table S5**. The ICD-10 codes used to identify SPL………………………………………...….…………22

**Table S6**. The various definitions of SPL among countries and international organizations……..........23

**Statistical analysis plan**..........................................................................................................................24

**OCM-Targeted Metabolomics in SPL**..................................................................................................26

**The Shanghai PreConception Cohort (SPCC) group**.........................................................................30

**References**...............................................................................................................................................31

**STROBE Statement**...............................................................................................................................32


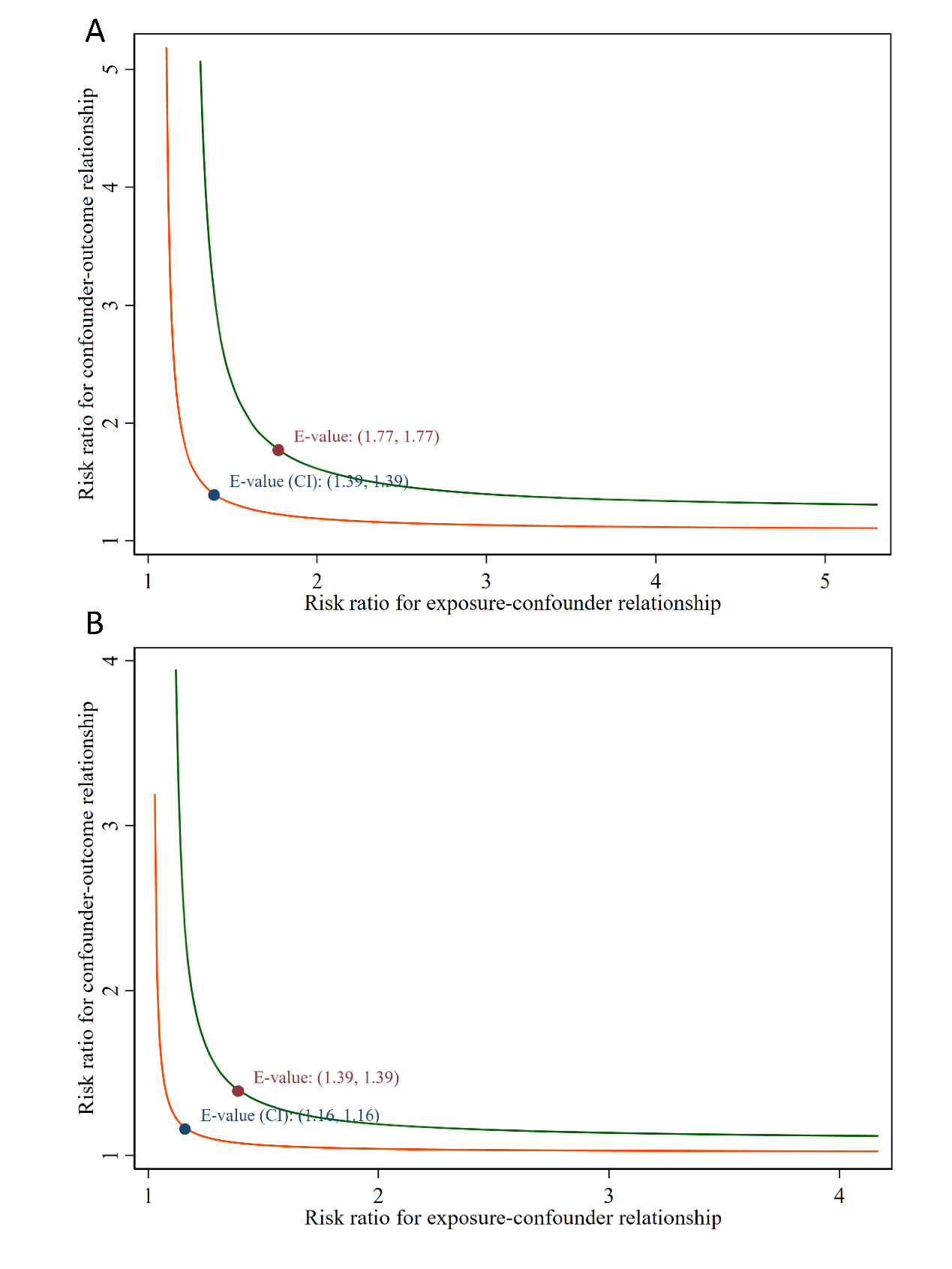


**Figure S1. E-value analysis for the observed association.** A, father; B, mother. The E-value quantifies the minimum strength of association, on the risk ratio scale, that an unmeasured confounder would need to have with both the exposure and outcome to fully explain away the observed association. The red point on the curve corresponds to the E-value for the point estimate, and the green point corresponds to the E-value for the confidence interval limit closest to the null. The curve shows the E-value (y-axis) for each possible risk ratio (x-axis).


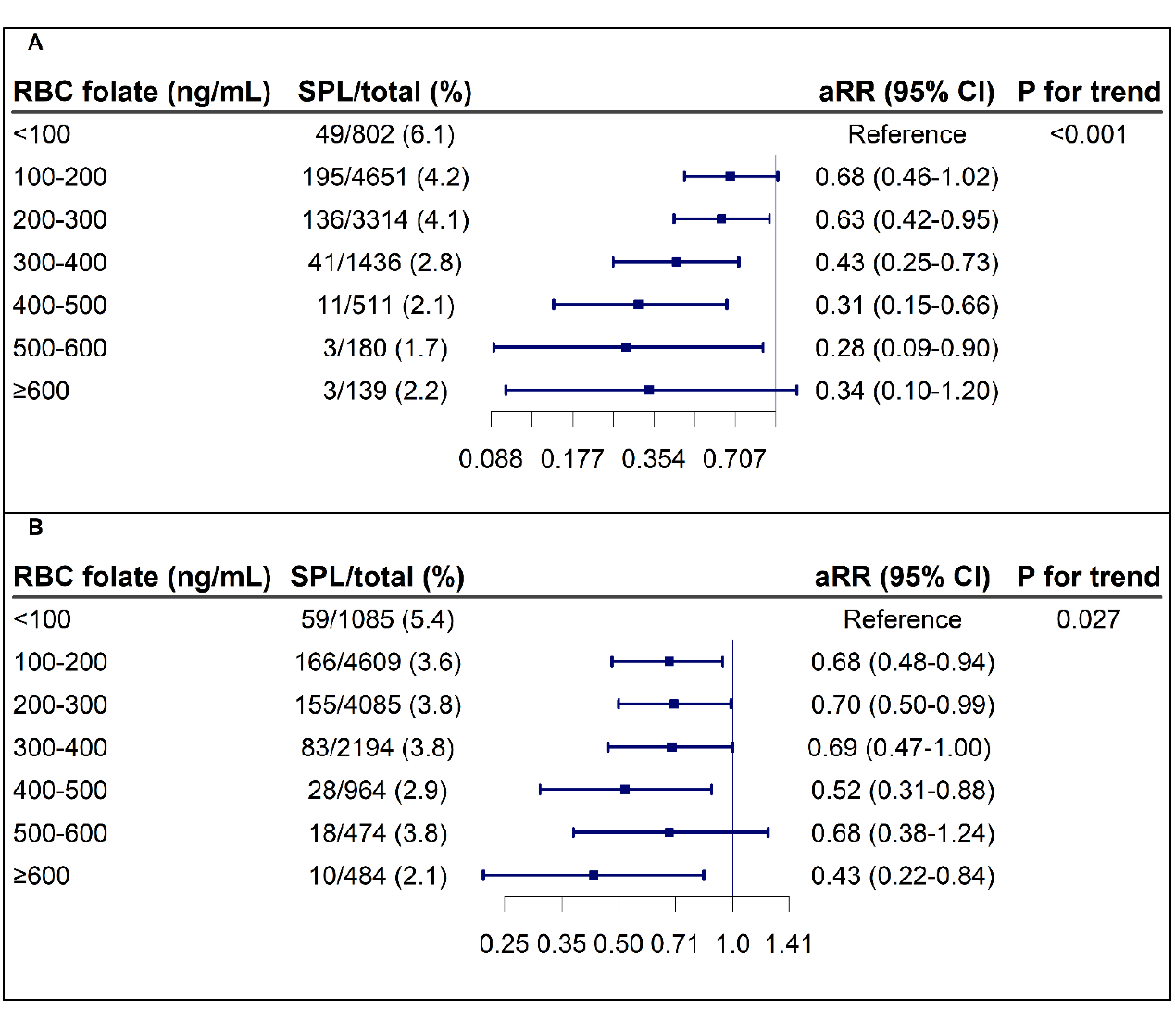


**Figure S2. Dose-response relationship between parental RBC folate levels before pregnancy with SPL risk.** A, father; B, mother. The axis was log-scaled. aRR, adjusted risk ratio; RBC, red blood cell; SPL, spontaneous pregnancy loss.


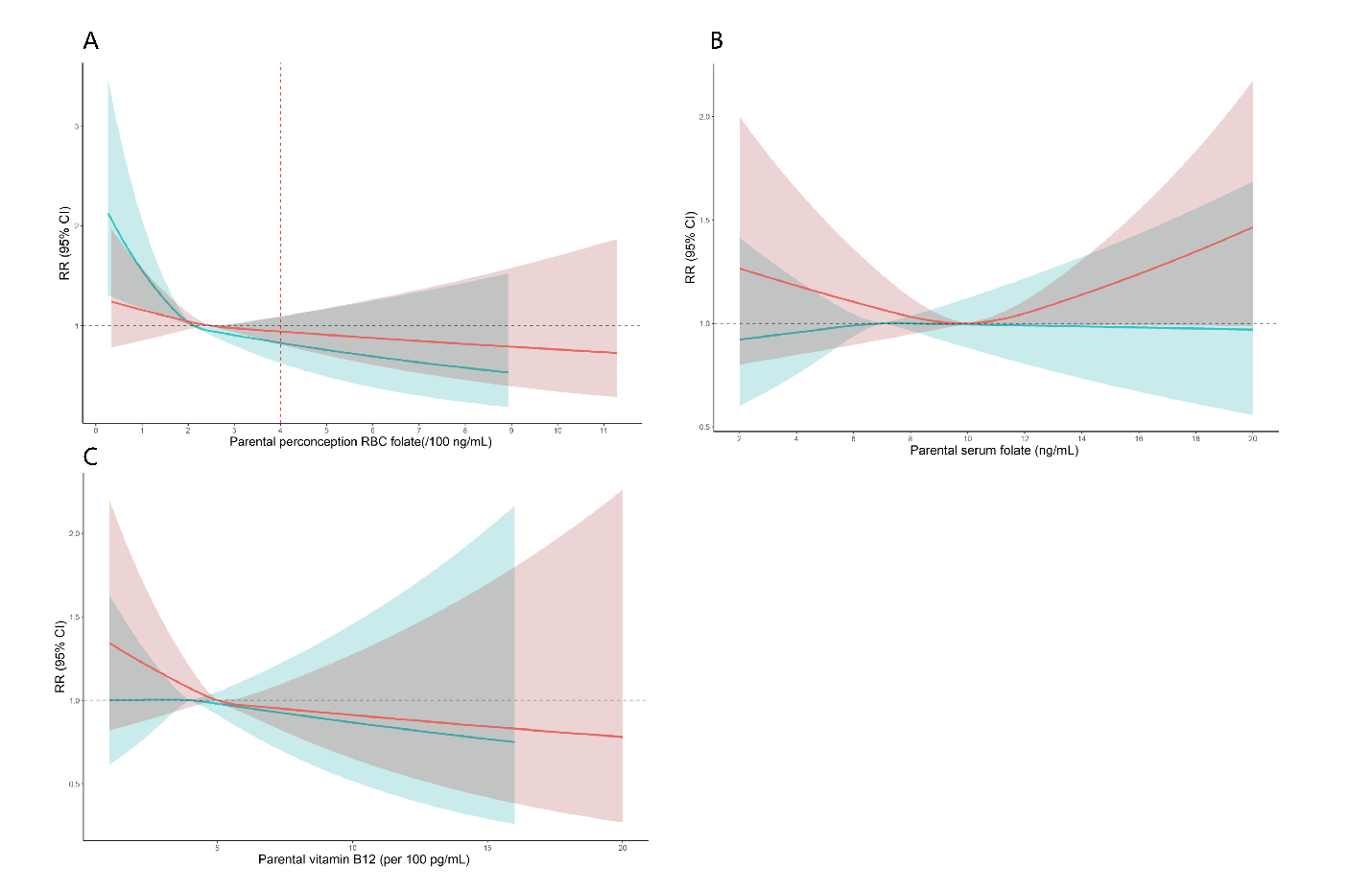


**Figure S3. Restricted cubic spline plots for the association between parental preconception OCM nutrient levels and SPL risk.**  A, RBC folate; B, serum folate; C, vitamin B12. Parental RBC folate, serum folate and vitamin B12 levels are coded with three knots located at the 25^th^, 50^th^, and 75^th^ percentiles of the distribution in the model. The median concentration was selected as the reference level. *P* values for non-linear of RBC folate, serum folate and vitamin B12 levels were: fathers 0.08, 0.73 and 0.82; mothers 0.68, 0.10, and 0.49. The red dash line indicates a target of 400 ng/mL for women recommended by the WHO. The light green and brown lines indicate estimated risk ratios of father and mothers, respectively. The light green and brown areas represent 95% CI. RBC, red blood cell.


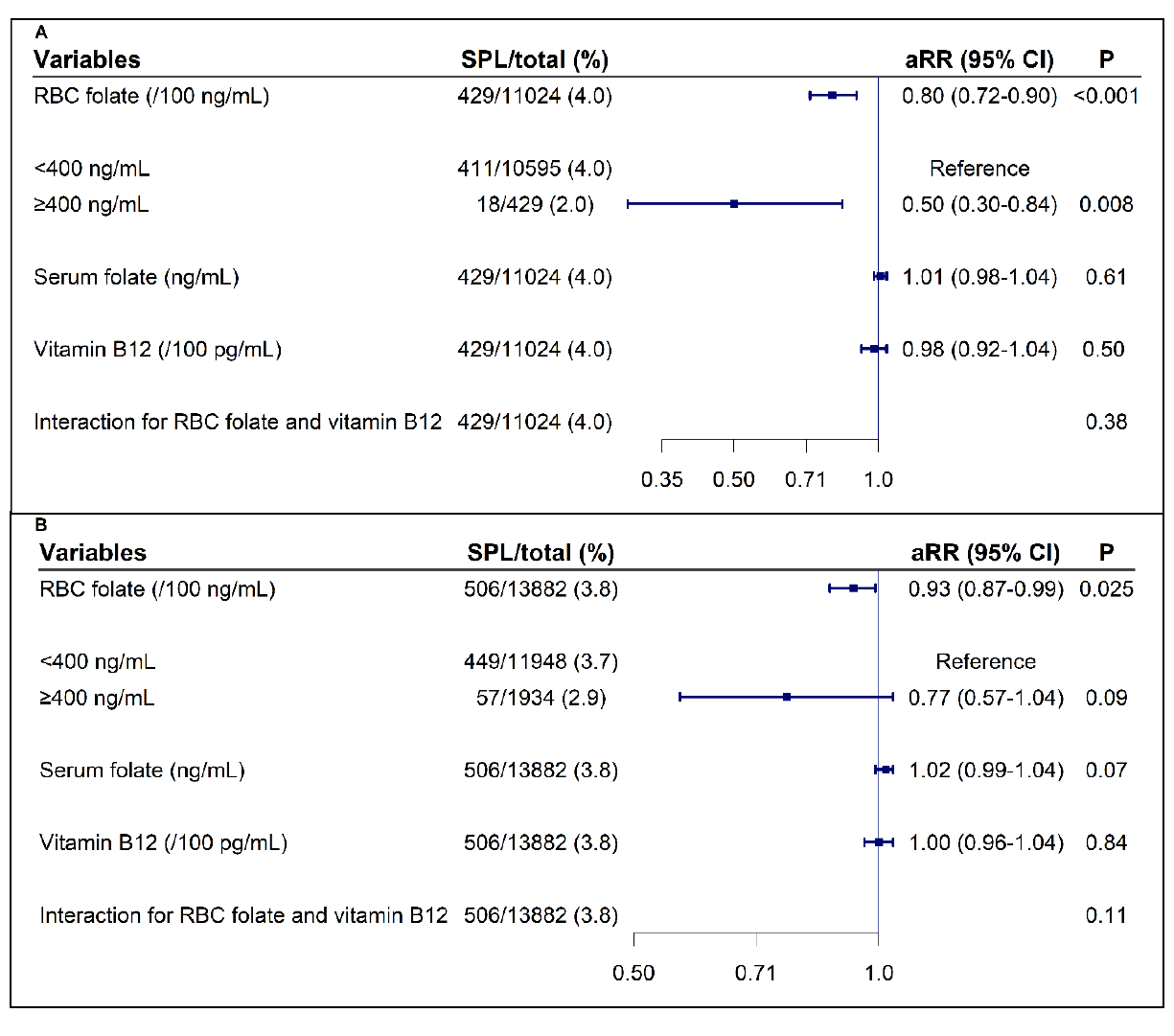


**Figure S4. Sensitivity analysis by defining SPL according to the Chinese guidelines.** A, father; B, mother. For the interaction analysis, RBC folate was categorized as a binary variable (<400 vs. ≥400 ng/mL), and vitamin B12 was treated as a continuous variable. The axis was log-scaled. aRR, adjusted risk ratio; RBC, red blood cell; SPL, spontaneous pregnancy loss.


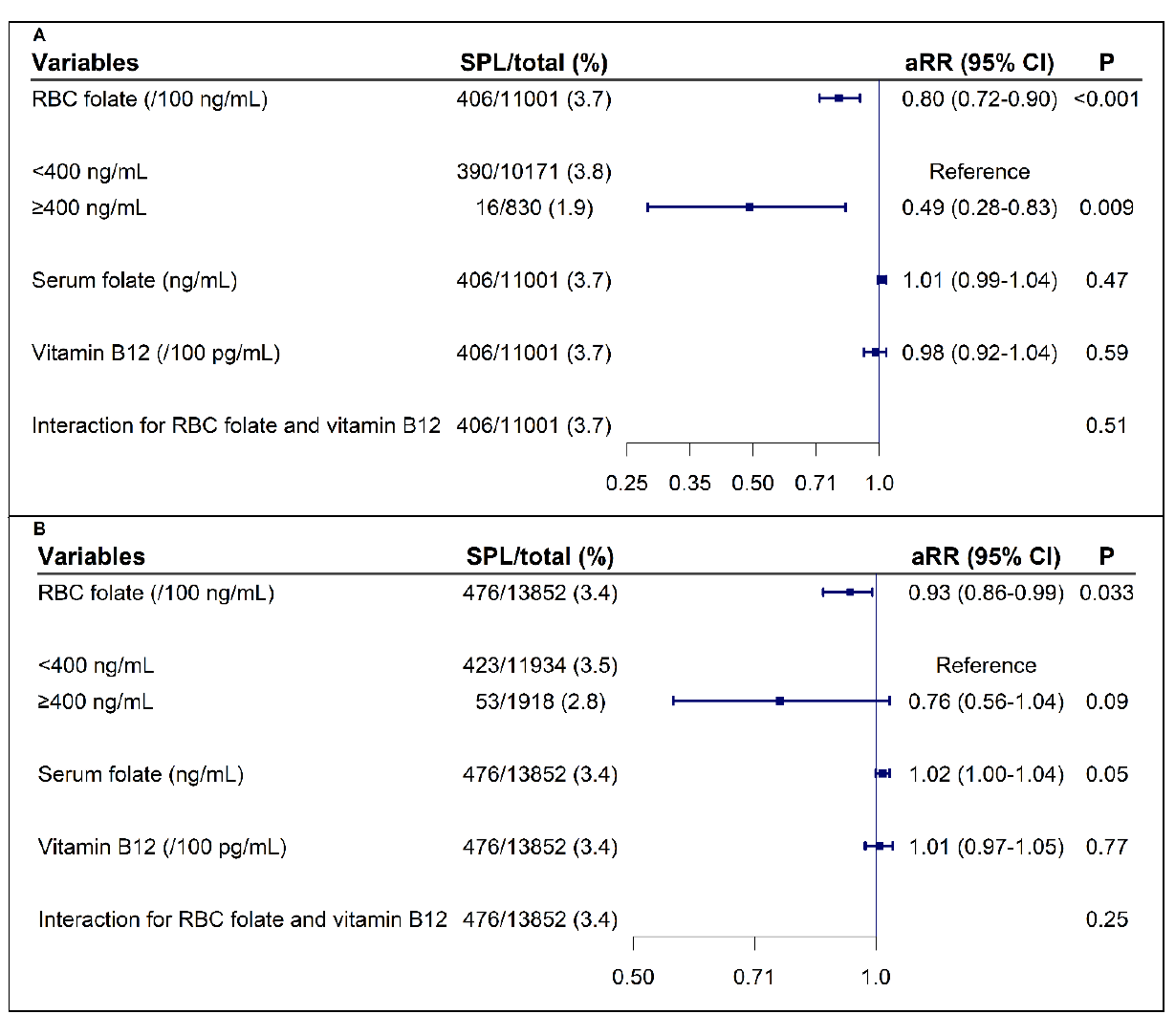


**Figure S5. Sensitivity analysis by defining SPL according to the European Society of Human Reproduction and Embryology guidelines.** A, father; B, mother. For the interaction analysis, RBC folate was categorized as a binary variable (<400 vs. ≥400 ng/mL), and vitamin B12 was treated as a continuous variable. The axis was log-scaled. aRR, adjusted risk ratio; RBC, red blood cell; SPL, spontaneous pregnancy loss.

**
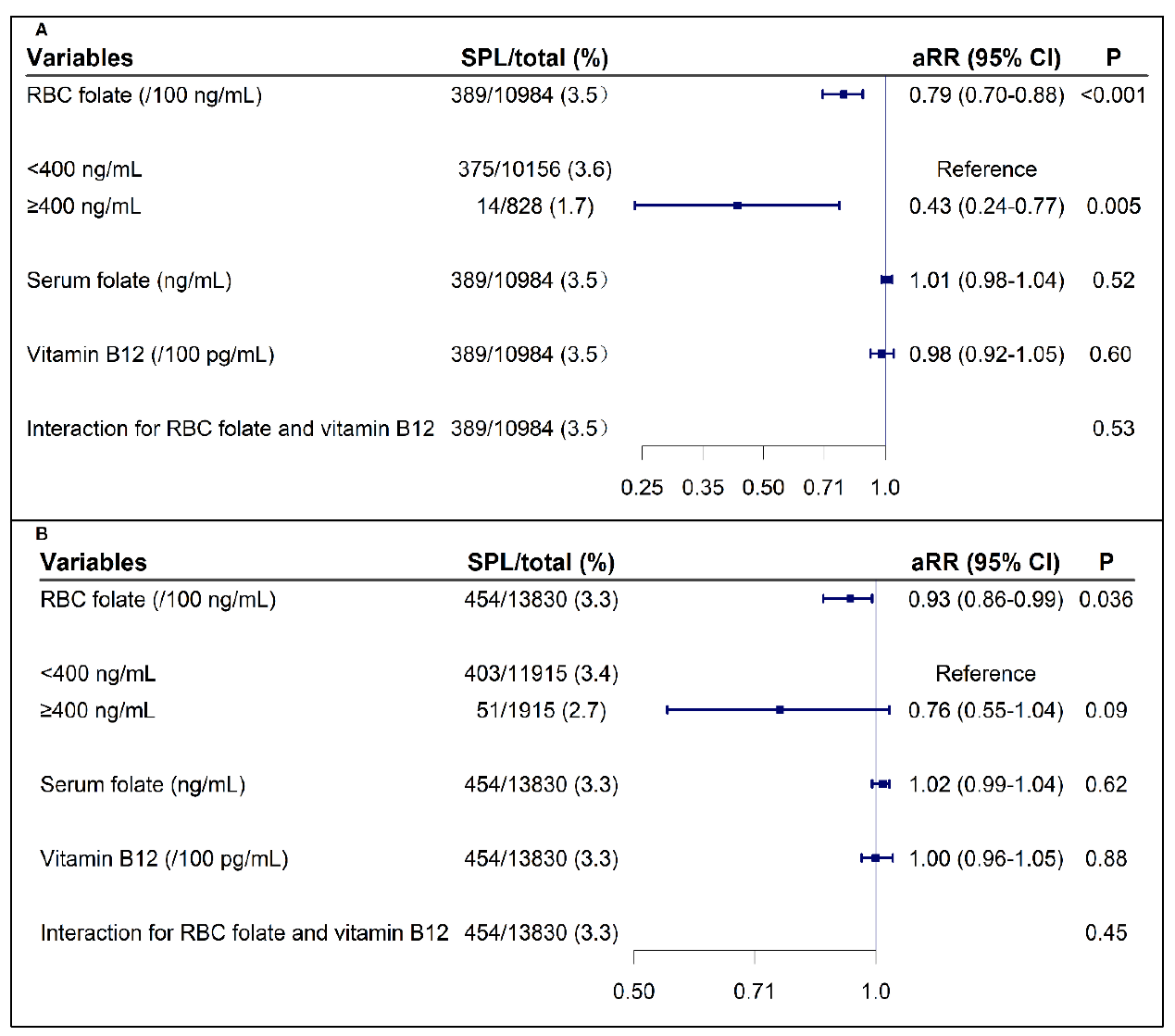
**

**Figure S6. Sensitivity analysis by defining SPL according to the WHO guidelines.** A, father; B, mother. For the interaction analysis, RBC folate was categorized as a binary variable (<400 vs. ≥400 ng/mL), and vitamin B12 was treated as a continuous variable. The axis was log-scaled. aRR, adjusted risk ratio; RBC, red blood cell; SPL, spontaneous pregnancy loss.


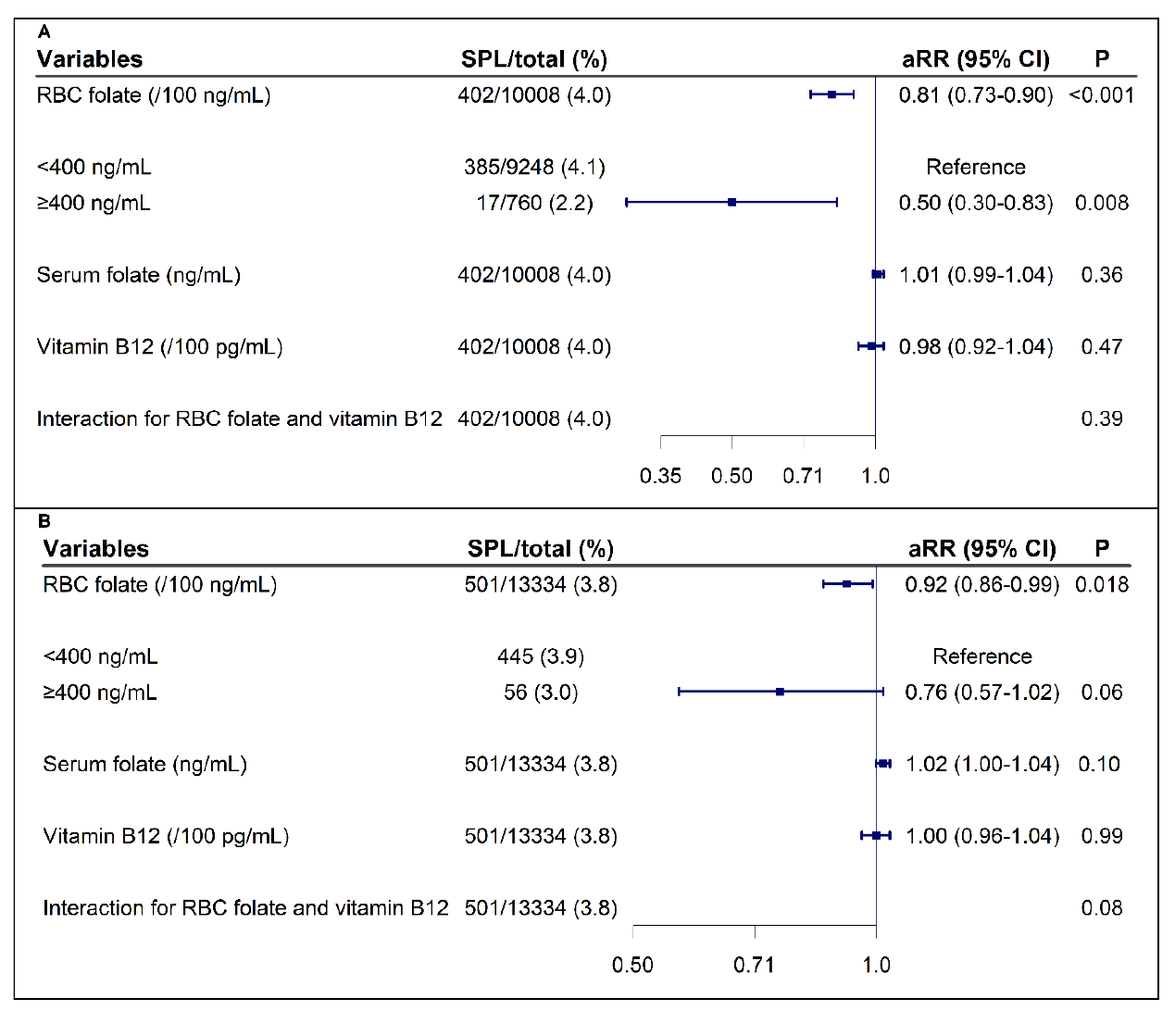


**Figure S7. Sensitivity analysis by excluding the 561 female and 1025 male participants with no blood samples.** A, father; B, mother. For the interaction analysis, RBC folate was categorized as a binary variable (<400 vs. ≥400 ng/mL), and vitamin B12 was treated as a continuous variable. The axis was log-scaled. aRR, adjusted risk ratio; RBC, red blood cell; SPL, spontaneous pregnancy loss.


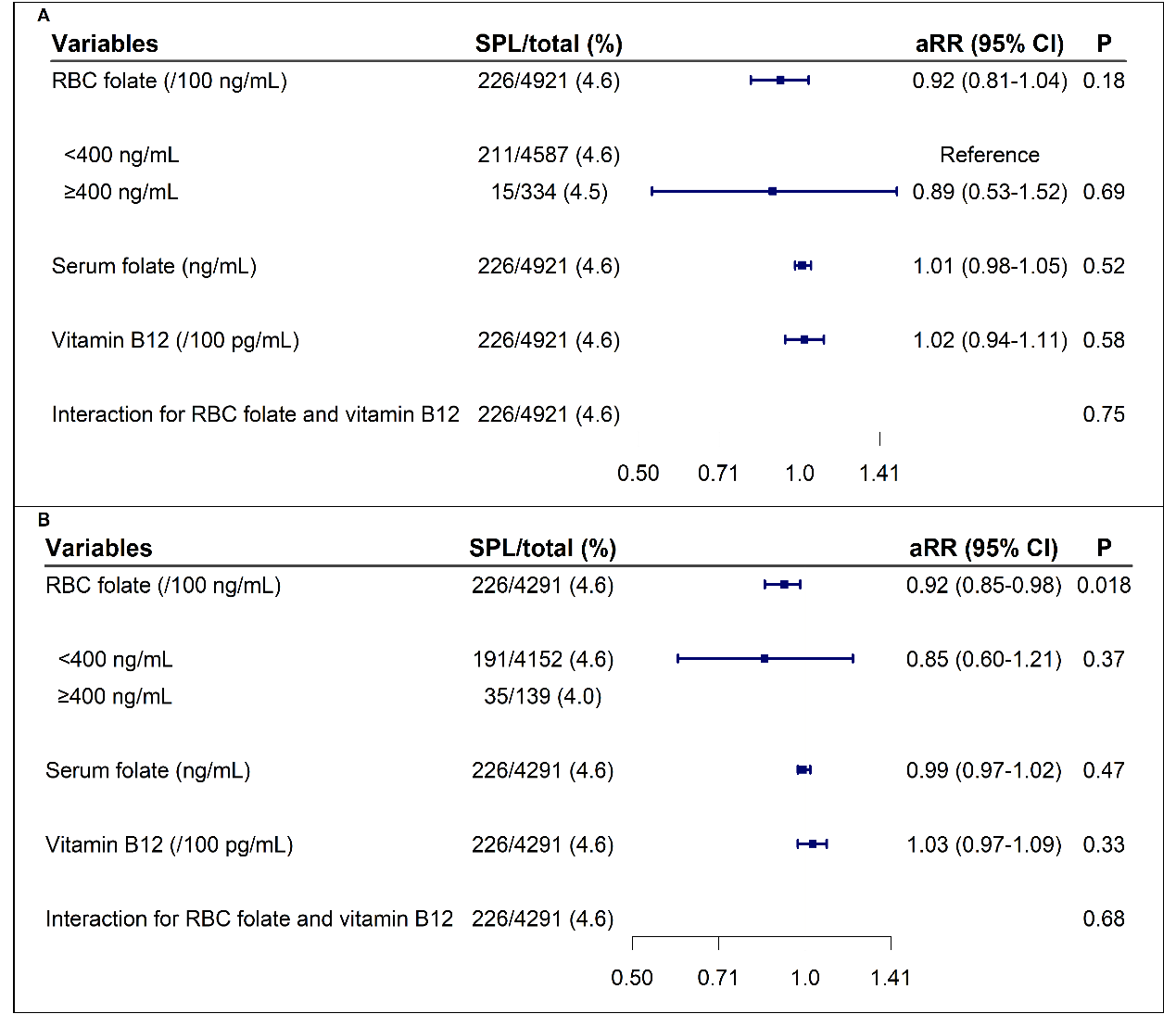


**Figure S8. Sensitivity analysis by** **addressing potential under‑ascertainment of SPL.** A, father; B, mother. The axis was log-scaled. For the interaction analysis, RBC folate was categorized as a binary variable (<400 vs. ≥400 ng/mL), and vitamin B12 was treated as a continuous variable. aRR, adjusted risk ratio; RBC, red blood cell; SPL, spontaneous pregnancy loss.


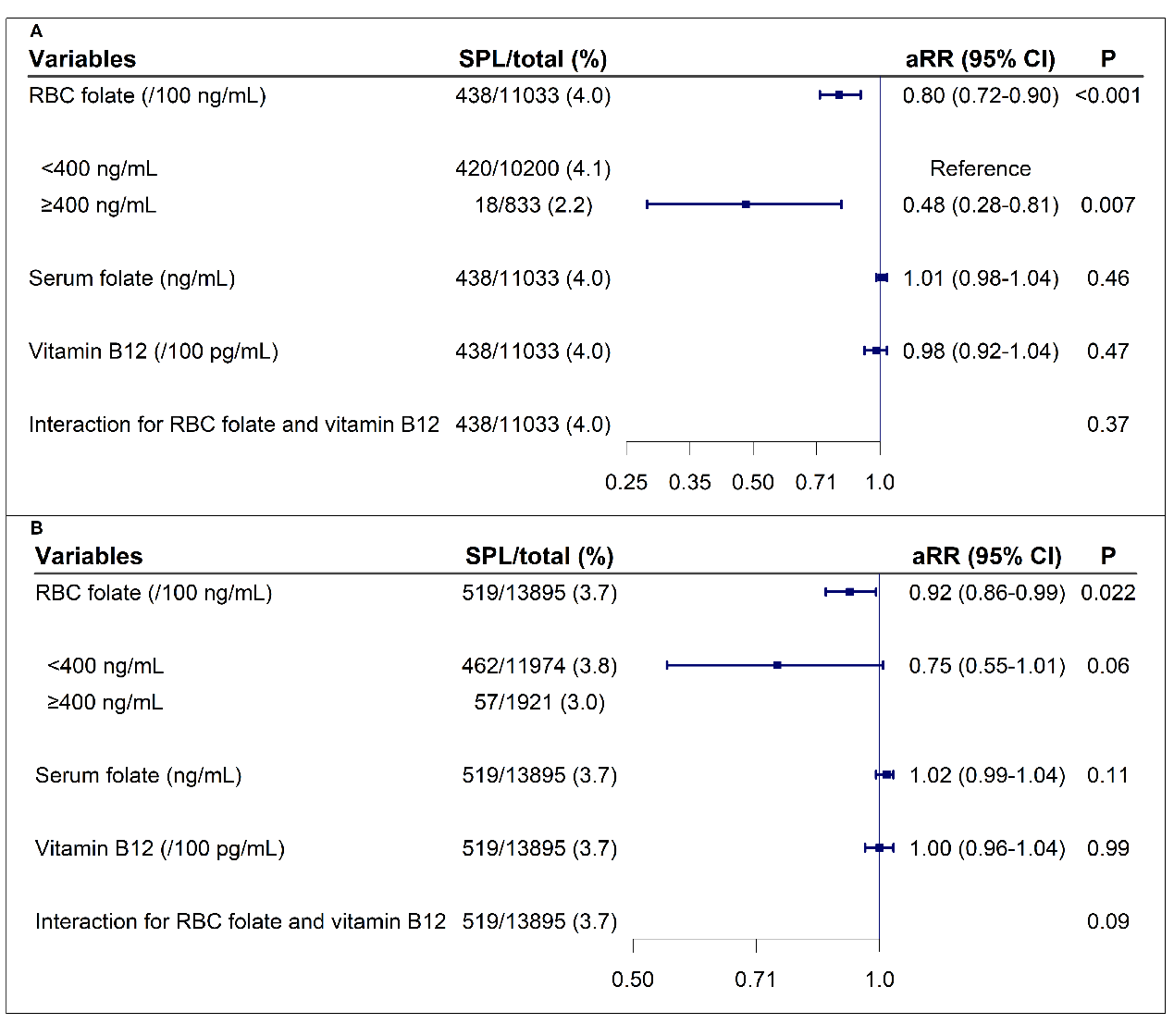


**Figure S9. Sensitivity analysis by adjusting covariates including parental education level.** A, father; B, mother. For the interaction analysis, RBC folate was categorized as a binary variable (<400 vs. ≥400 ng/mL), and vitamin B12 was treated as a continuous variable. The axis was log-scaled. aRR, adjusted risk ratio; RBC, red blood cell; SPL, spontaneous pregnancy loss.


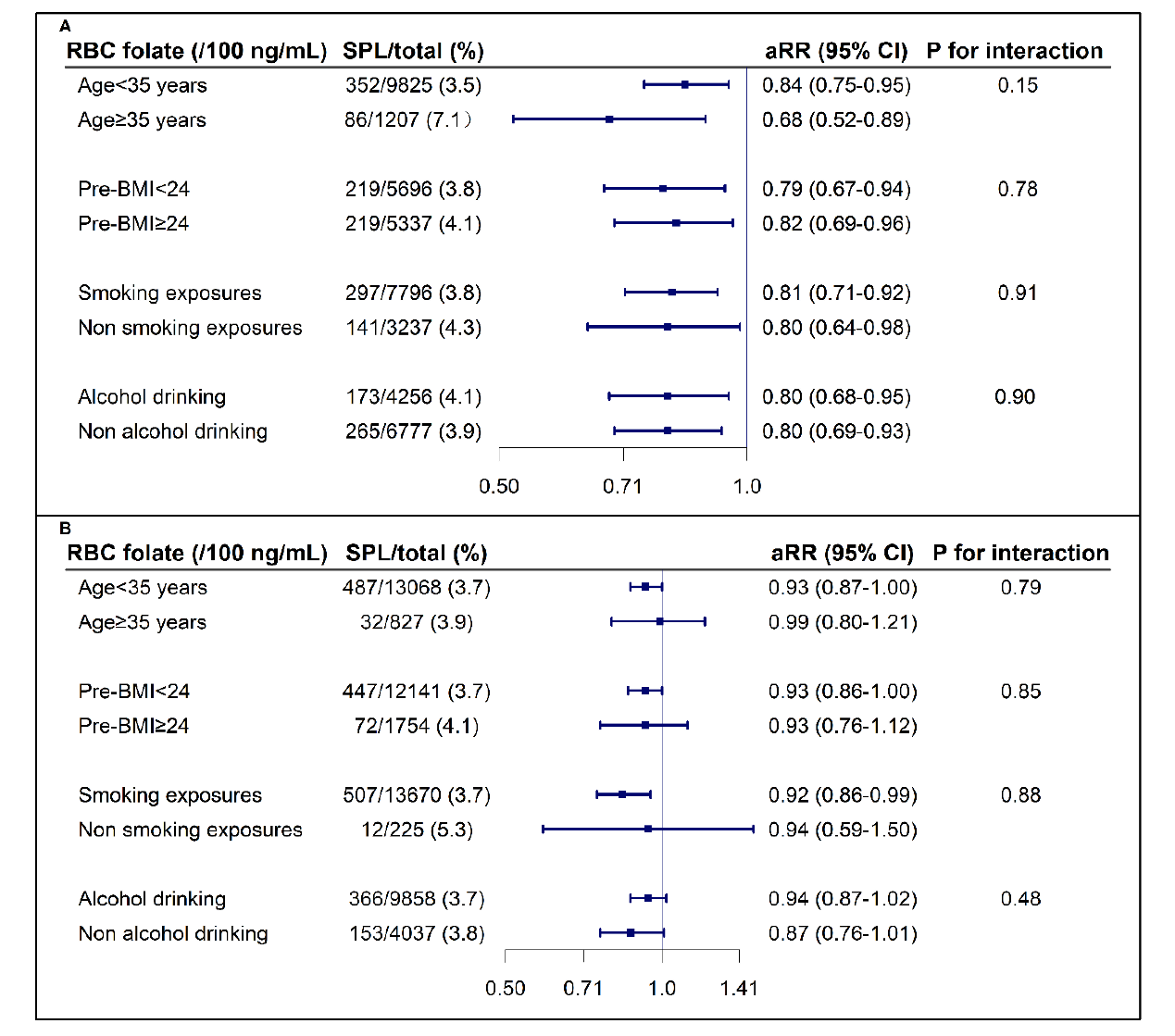


**Figure S10. Subgroup analysis based on age group (<35 years, ≥35 years), BMI (<24 years, ≥35 years), smoking exposures (Yes, No) and alcohol drinking (Yes, No).** A, father; B, mother. For the interaction analysis, RBC folate was treated as a continuous variable and the other subgroups were categorized as a binary variable, respectively. The axis was log-scaled. aRR, adjusted risk ratio; Pre-BMI, preconception body mass index; RBC, red blood cell; SPL, spontaneous pregnancy loss.


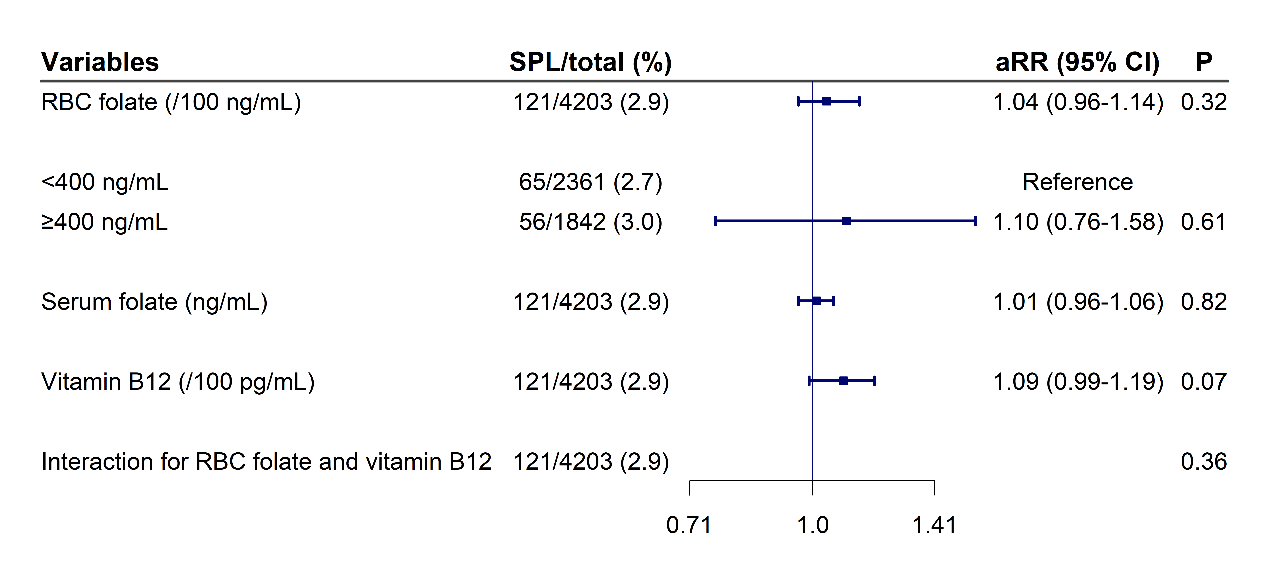


**Figure S11. Associations of maternal OCM-related nutrient levels at early gestation with SPL risk.** For the interaction analysis, RBC folate was categorized as a binary variable (<400 vs. ≥400 ng/mL), and vitamin B12 was treated as a continuous variable. The axis was log-scaled. RBC, red blood cell; RR, risk ratio; SPL, spontaneous pregnancy loss.


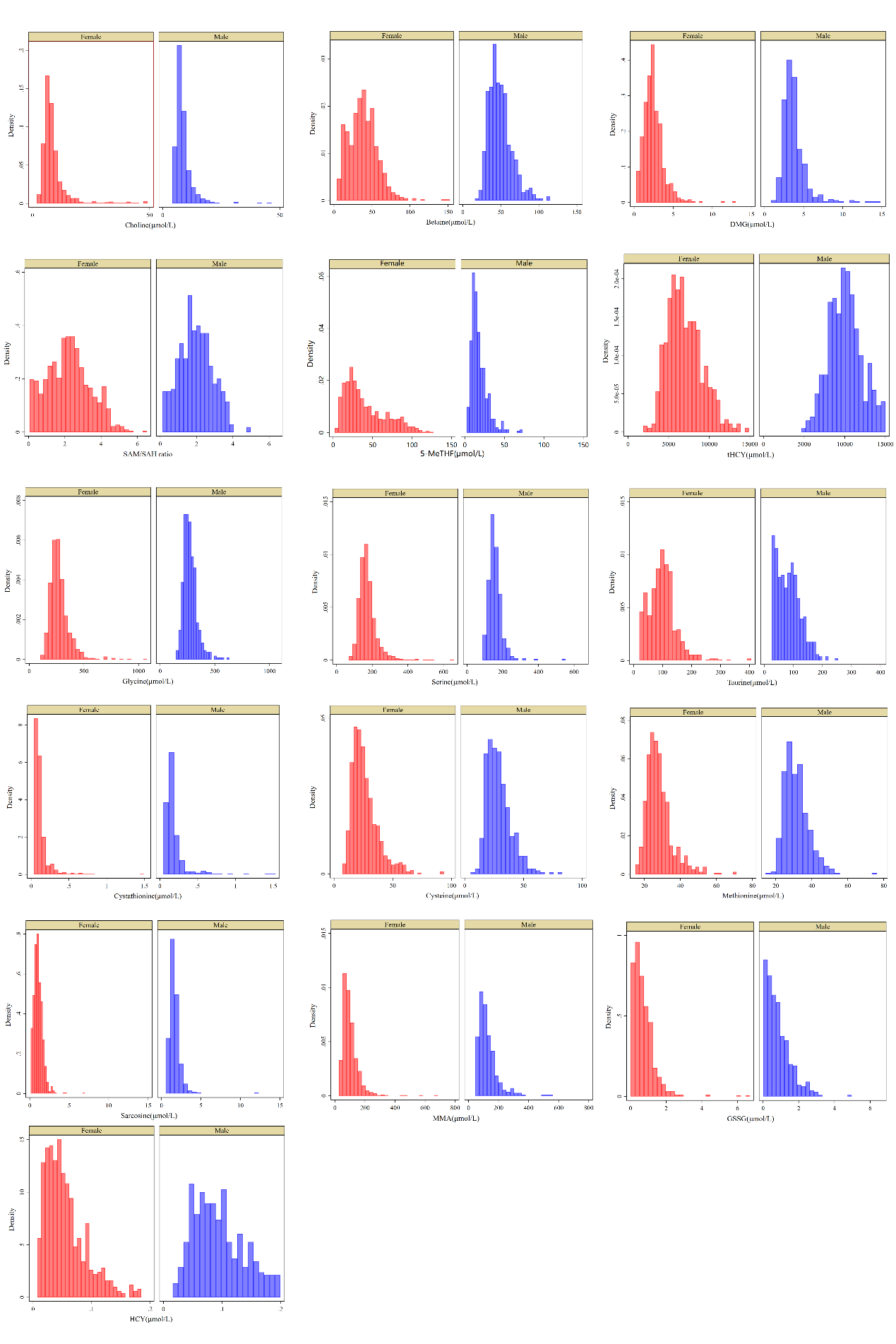


**Figure S12. The distribution of serum OCM metabolites between preconception fathers and mothers.** DMG, dimethylglycine; HCY, homocysteine; GSSG, glutathione oxidized; GSH, glutathione reduced; MMA, methylmalonic acid; OCM, one-carbon metabolism; SAM, S-Adenosly Methionine; SAH, S-Adenosyl homocysteine; 5-MeTHF, 5-Methyltetrahydrofolate.


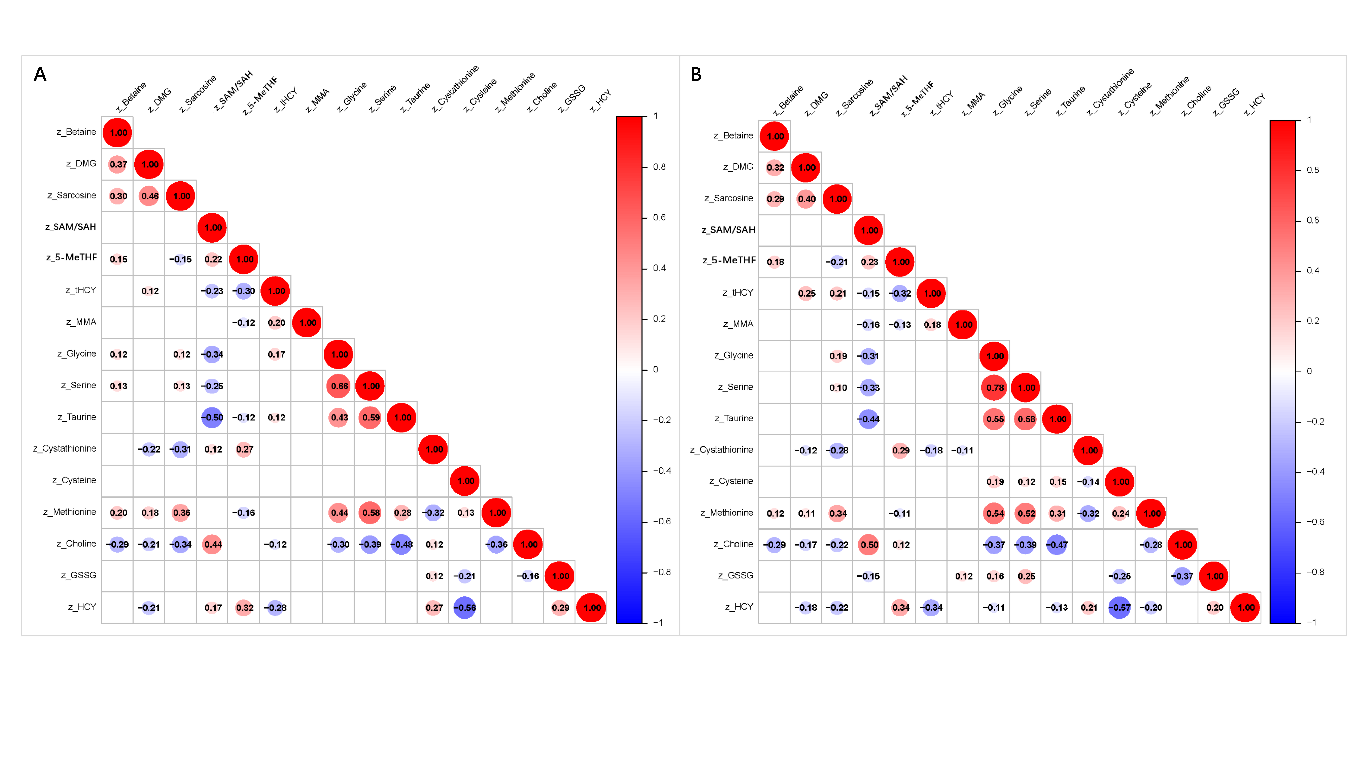


**Figure S13. Correlation heatmap for preconception parental OCM metabolites.** A, SPL cases; B, controls. The values in the figure represent the correlation coefficient β values with *P*<0.05. All metabolites were normalized into z-scores. DMG, dimethylglycine; HCY, homocysteine; GSSG, glutathione oxidized; GSH, glutathione reduced; MMA, methylmalonic acid; OCM, one-carbon metabolism; SAM, S-Adenosly Methionine; SAH, S-Adenosyl homocysteine; 5-MeTHF, 5-methyltetrahydrofolate

**
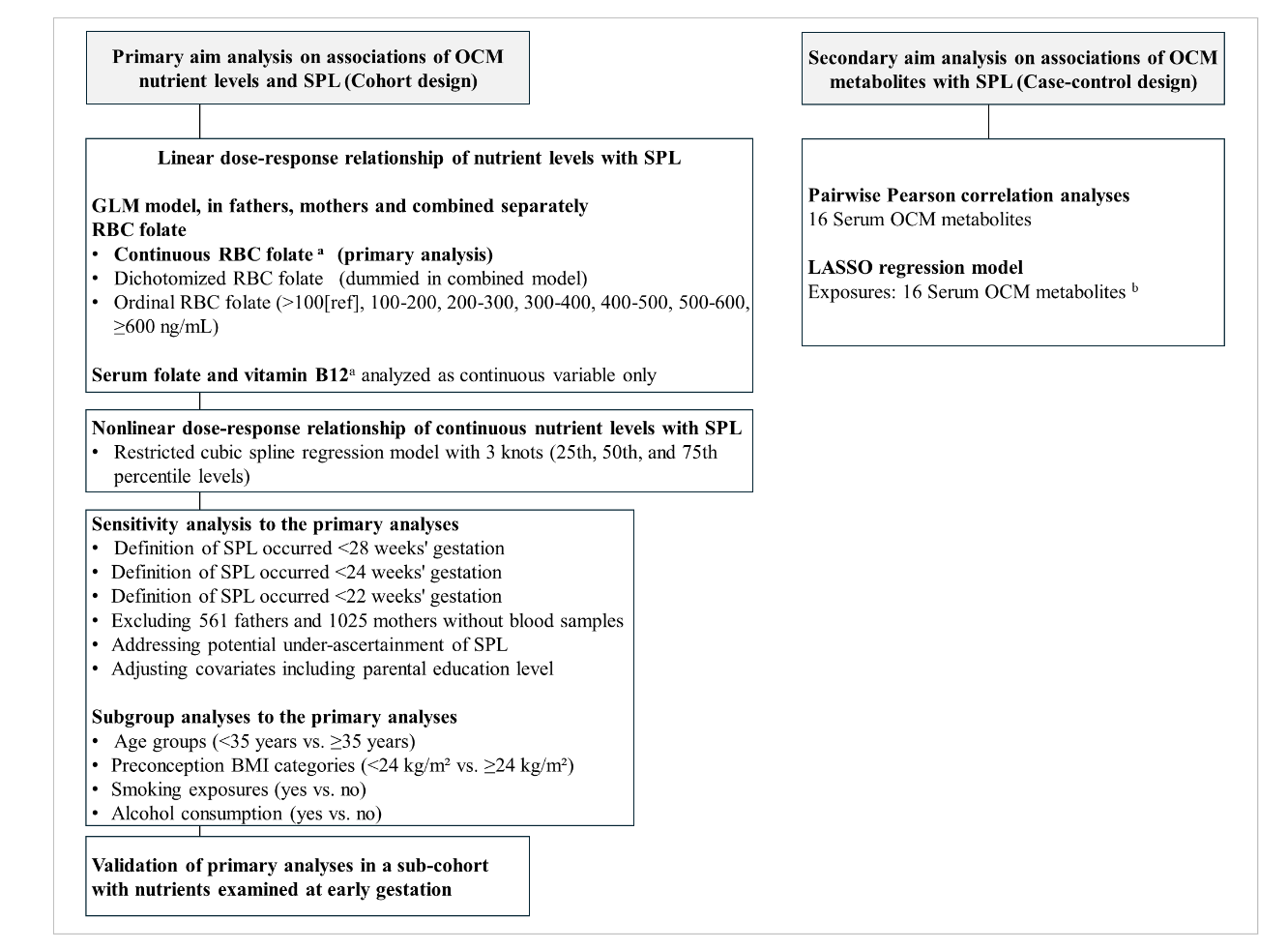
**

**Figure S14. Statistical analysis plan.** a, rescaled per 100; b, all metabolites were log-transformed to approximate normality and standardized to z-scores prior to analysis. Parental RBC folate with the SPL risk. Adjusted covariates included paternal and maternal age, preconception BMI, smoking status, drinking status, gravidity, history of adverse pregnancy outcomes, and the duration (months) between enrollment and conception in the GLM model. Age preconception BMI, smoking and drinking status were adjusted in weighted logistic regression model and LASSO regression model. GLM, generalized linear models; SPL, spontaneous pregnancy loss.

**Table S1. Baseline characteristics of SPL and non-SPL population**

| Variables | Fathers | |  | Mothers | | *P* |
| --- | --- | --- | --- | --- | --- | --- |
|  | SPL | Non-SPL | *P* | SPL | Non-SPL |  |
| N | 438 | 10595 |  | 519 | 13376 |  |
| Age (years), mean (SD) | 31.2(4.5) | 30.0(3.9) | <0.001 | 29.6(3.6) | 28.9(3.5) | <0.001 |
| <35 years, n (%) | 351(80.1) | 9470(89.4) | <0.001 | 478(92.1) | 12588(94.1) | 0.06 |
| ≥35 years, n (%) | 86(19.6) | 1121(10.6) |  | 41(7.9) | 785(5.9) |  |
| Missing, n (%) | 1(0.2) | 4(<0.1) |  | 0 | 3(<0.1) |  |
| Preconception BMI (Kg/m^2^), mean (SD) | 24.2(3.3) | 24.0(3.3) | 0.25 | 21.2(2.7) | 21.0(2.8) | 0.17 |
| <24 Kg/m^2^, n (%) | 181(41.3) | 4454(42.0) | 0.90 | 441(85.0) | 11610(86.8) | 0.50 |
| ≥24 Kg/m^2^, n (%) | 162(37.0) | 3931(37.1) |  | 69(13.3) | 1662(12.4) |  |
| Missing, n (%) | 95(21.7) | 2210(20.9) |  | 9(1.7) | 104(0.8) |  |
| Smoking exposure, n (%) | 137(31.3) | 3070(29.0) | 0.24 | 12(2.3) | 210(1.6) | 0.18 |
| Missing, n (%) | 7(1.6) | 74(0.7) |  | 8(1.5) | 83(0.6) |  |
| Drinking, n (%) | 262(59.8) | 6444(60.8) | 0.63 | 147(28.3) | 3849(28.8) | 0.89 |
| Missing, n (%) | 3(0.7) | 96(0.9) |  | 8(1.5) | 132(0.9) |  |
| Gravidity, n (%) |  |  |  |  |  |  |
| 1 | 243(55.2) | 6368(60.1) | 0.023 | 287(55.3) | 8030(60.0) | 0.009 |
| >1 | 167(38.1) | 3468(32.7) |  | 204(39.3) | 4478(33.5) |  |
| Missing, n (%) | 28(6.4) | 759(7.2) |  | 28(5.4) | 868(6.5) |  |
| Abnormal pregnancy history, n (%) | 141(32.2) | 4381(41.3) | <0.001 | 143(27.6) | 4519(33.8) | 0.003 |
| Missing, n (%) | 0 | 0 |  | 0 | 0 |  |
| RBC folate (ng/mL), median (IQR) | 187.0(132.2, 240.8) | 196.8(144.0, 270.6) | 0.002 | 224.9(167.0., 323.3) | 222.7(162.5, 316.3) | 0.47 |
| <400 ng/mL, n (%) | 357 (81.5) | 7820 (73.8) | 0.009 | 421(81.2) | 10209 (76.3) | 0.07 |
| ≥400 ng/mL, n (%) | 15 (3.4) | 652 (6.2) |  | 50(9.6) | 1597 (12.0) |  |
| Missing, n (%) | 66 (15.1) | 2123 (20.0) |  | 48(9.2) | 1570 (11.7) |  |
| Serum folate (nmol/L), median (IQR) | 6.4 (4.8, 9.3) | 6.1 (4.5, 8.7) | 0.030 | 9.9(6.9, 14.1) | 9.3(6.4, 13.3) | 0.033 |
| Missing, n (%) | 53 (12.1) | 1388 (13.1) |  | 46(8.9) | 1442(10.8) |  |
| Vitamin B12 (pg/mL), median (IQR) | 396.0 (308.0, 517.0) | 394.0(307.0, 507.0) | 0.94 | 486.0(379.0, 617.0) | 496.0(378.0, 640.0) | 0.49 |
| Deficiency (<200 pg/mL), n (%) | 20 (4.6) | 362 (3.4) | 0.28 | 11 (2.1) | 281 (2.1) | 0.61 |
| Missing, n (%) | 57(13.0) | 1410(13.3) |  | 38 (7.3) | 1156 (8.6) |  |
| Duration between enrollment and conception (month), median (IQR) | 14.9(14.8) | 12.4(11.8) | <0.001 | 15.1(14.8) | 12.5(12.1) | <0.001 |
| Missing, n (%) | 90 (20.5) | 629(5.9) |  | 92(17.7) | 1456(10.9) |  |

Continuous data were summarized as mean (SD) or median (IQR), and categorical data were displayed as percentages. BMI, body mass index; IQR, interquartile range; RBC, red blood cell; SD, standard deviation; SPL, spontaneous pregnancy loss

**Table S2.** **Characteristics of** **the mothers with OCM nutrient levels at early gestation between SPL and non-SPL (N=4203)**

| Variables | Mothers | |
| --- | --- | --- |
|  | SPL | Non-SPL |
| N | 121 | 4082 |
| Age (years), mean (SD) | 28.5 (3.7) | 27.9 (3.2) |
| <35 years, n (%) | 113 (93.4) | 3954 (96.9) |
| ≥35 years, n (%) | 8 (6.6) | 128 (3.1) |
| Preconception BMI (Kg/m^2^), mean (SD) | 21.2 (2.6) | 20.8 (2.6) |
| <24 Kg/m^2^, n (%) | 105 (86.8) | 3616 (88.6) |
| ≥24 Kg/m^2^, n (%) | 16 (13.2) | 464 (11.4) |
| Missing, n (%) | 0 | 2 (0.05) |
| Smoking exposure, n (%) | 1 (0.8) | 29 (0.7) |
| Missing, n (%) | 3 (2.5) | 26 (0.6) |
| Drinking, n (%) | 34 (28.1) | 1011 (24.8) |
| Missing, n (%) | 1 (0.8) | 23 (0.6) |
| Gravidity, n (%) |  |  |
| 1 | 2241 (54.9) | 58 (47.9) |
| >1 | 1356 (33.2) | 50 (41.3) |
| Missing, n (%) | 485 (11.9) | 13 (10.7) |
| Abnormal pregnancy history, n (%) | 113 (93.4) | 1265 (31.0) |
| OCM-related nutrient levels at early gestation | | |
| RBC folate (ng/mL), median (IQR) | 244.1 (172.2, 345.1) | 235.6 (170.6, 339.2) |
| <400 ng/mL, n (%) | 95 (78.5) | 3252 (79.7) |
| ≥400 ng/mL, n (%) | 22 (18.2) | 676 (16.6) |
| Missing, n (%) | 4 (3.3) | 154 (3.8) |
| Serum folate (nmol/L), median (IQR) | 9.4 (7.0, 14.8) | 9.3 (6.5, 13.3) |
| Missing, n (%) | 5 (4.1) | 90 (2.2) |
| Vitamin B12 (pg/mL), median (IQR) | 467.0 (373.0, 587.5) | 485.0 (373.0, 620.0) |
| Missing, n (%) | 6 (4.9) | 86 (2.1) |
| Duration between enrollment and conception (month), median (IQR) | 11.7 (10.8) | 10.4 (8.9) |
| Missing, n (%) | 50 (1.2) | 5 (4.1) |

Continuous data were summarized as mean (SD) or median (IQR), and categorical data were displayed as percentages. BMI, body mass index; IQR, interquartile range; RBC, red blood cell; SD, standard deviation.

**Table S3. Baseline characteristics of participants in the OCM targeted metabolomics study stratified by SPL cases and controls**

|  | Fathers | | Mothers | |
| --- | --- | --- | --- | --- |
| Variables | SPL | Non-SPL | SPL | Non-SPL |
| N | 147 | 173 | 150 | 200 |
| Age, mean (SD), y | 31.4 (4.6) | 30.3 (4.0) | 29.6 (3.9) | 29.1 (3.7) |
| <35 years | 113 (76.9) | 154 (89.0) | 135 (90.0) | 185 (92.5) |
| ≥35 years | 34 (23.1) | 19 (11.0) | 15 (10.0) | 15 (7.5) |
| Preconception BMI, mean (SD), Kg/m^2^ | 24.33 (3.3) | 23.87 (2.9) | 21.12 (2.6) | 21.46 (3.0) |
| <24 Kg/m^2 a^ | 73 (49.7) | 79 (45.7) | 133 (88.7) | 164 (82.0) |
| ≥24 Kg/m^2^ | 74 (50.3) | 94 (54.3) | 17 (11.3) | 36 (18.0) |
| Smoking exposure | 38 (25.9) | 54 (31.2) | 2 (1.3) | 45 (22.5) |
| Drinking | 88 (59.9) | 106 (61.3) | 41 (27.3) | 57 (28.5) |
| RBC folate, median (IQR), ng/mL | 184.41 (135.13, 235.48) | 183.57 (136.51, 251.72) | 235.30 (174.15, 320.58) | 230.16 (168.44, 317.48) |

Continuous data were summarized as mean (SD) or median (IQR), and categorical data were displayed as percentages. Unless otherwise indicated, data were expressed as No. (%) of subjects. BMI, body mass index; IQR, interquartile range; RBC, red blood cell; SD, standard deviation.

a, the BMI was categorized according to the definitions for the Chinese population

**Table S4. The estimated coefficients for LASSO regression between parental preconception RBC folate, OCM metabolites and SPL risk.**

|  | Fathers | | Mothers | |
| --- | --- | --- | --- | --- |
| Exposures | β coefficients | Bootstrap SE | β coefficients | Bootstrap SE |
| OCM metabolites |  |  |  |  |
| Choline | 0 | - | 0 | - |
| GSSG | 0.17 | 0.11 | 0 | - |
| Cystathionine | 0.06 | 0.08 | 0 | - |
| Hcy | 0 | - | 0 | - |
| Betaine | 0.09 | 0.11 | 0.02 | 0.08 |
| DMG | 0 | - | -0.19 | 0.15 |
| Sarcosine | 0 | - | 0.05 | 0.11 |
| SAM/SAH ratio | 0 | - | 0.20 | 0.10 |
| tHCY | 0 | - | 0 | - |
| MMA | 0.12 | 0.10 | 0 | - |
| Glycine | 0 | - | 0 | - |
| Serine | 0 | - | 0 | - |
| Taurine | -0.09 | 0.11 | -0.03 | 0.08 |
| Cysteine | 0 | - | 0.09 | 0.10 |
| Methionine | 0 | - | 0 | - |
| RBC folate | 0 | - | 0 | - |

Preconception age, preconception BMI, smoking and drinking status were treated as covariates, and preconception RBC folate and OCM metabolites were treated as exposures in the LASSO regression model. To explore the associations among RBC folate, OCM metabolites, and SPL, we substituted 5-MeTHF with RBC folate in the analysis, given that 5-MeTHF constitutes the predominant metabolic component of RBC folate. GSSG, oxidized glutathione; Hcy, homocysteine; MMA, methylmalonic acid; SAM, S-adenosylmethionine; SAH, S-adenosylhomocysteine.

**Table S5. The ICD-10 codes used to identify SPL.**

|  | **ICD-10** |
| --- | --- |
| Ectopic pregnancy | O00 |
| Tubal pregnancy | O00.1 |
| Ectopic pregnancy, unspecified | O00.9 |
| Spontaneous abortion | O03 |
| Unspecified abortion | O06 |
| Failed attempted abortion | O07 |

ICD-10, International Classification of Diseases 10th Revision.

**Table S6. The various definitions of SPL among countries and international organizations.**

| **Countries or international organizations** | **Definitions for SPL** |
| --- | --- |
| China^1^ | Fetal death occurs from the time of conception until 28 weeks of gestation. |
| European Society of Human Reproduction and Embryology^2^ | Fetal death occurs from the time of conception until 24 weeks gestation. |
| WHO^3, 4^ | Fetal death occurs from the time of conception until 22 weeks gestation. |

SPL, spontaneous pregnancy loss; WHO, World Health Organization.

**Statistical analysis plan**

**1. Primary aim**

To quantify the associations of parental preconception RBC folate, serum folate, and vitamin B12 concentrations with subsequent SPL risk.

**2. Secondary aim**

To evaluate the associations of parental preconception serum OCM metabolites with SPL.

**3. Study design**

In the primary aim analysis, we applied a prospective cohort including 11,033 couples and 2,862 single mothers planning for pregnancy drawn from the ongoing SPCC. To validate the primary analysis, we examined maternal RBC folate, serum folate, and vitamin B12 concentrations during early gestation in a sub-cohort (n=4203). For the secondary aim, we performed targeted OCM metabolomics in an exploratory nested case-control subcohort (150 SPL cases and 200 controls).

**4. Statistical analysis**

**4.1 Descriptive Analysis**

Continuous variables were reported as mean (standard deviation [SD]) when normally distributed and as median (inter-quartile ranges [IQRs]) where not. The normality was assessed by visual inspection of the frequency histograms. Categorical variables were summarized as numbers and percentages. For comparisons between the SPL and the non-SPL group were performed for continuous variables using two-tailed unpaired Student’s *t*-tests or Mann-Whitney U tests depending on normality, while categorical variables were compared with a chi-square test.

**4.2 Primary aim analyses**

Generalized linear models (GLM) with binomial family and log link function fitted by iterated reweighted least squares method was used to estimate the risk ratios (RRs) and 95% confidence intervals (CIs) for the associations of paternal and maternal preconception RBC folate (continuous or categorical), serum folate (continuous), and vitamin B12 (continuous) with subsequent SPL risk, respectively ^5^. We initially treated paternal and maternal concentrations of these three nutrients as continuous or categorical variables in separate models adjusted for corresponding confounders, followed by a combined model ^6^. Continuous RBC folate and vitamin B12 levels were rescaled per 100. To facilitate clinical interpretation, we repeated the association analysis based on the dummied statuses of paternal, maternal and both achieving the WHO recommended cutoff of RBC folate^7^. Further, we used a restricted cubic spline (RCS) regression model with 3 knots (25^th^, 50^th^, and 75^th^ percentile levels) fitted in R ("rms" package) to assess the potential nonlinear dose-response relationship of continuous nutrient levels with the SPL risk. We used DAG to determine the smallest set of covariates for adjustment, including paternal and maternal age, preconception BMI, smoking status, drinking status, gravidity, history of adverse pregnancy outcomes, and the duration (months) between enrollment and conception. To identify potential threshold effects of RBC folate on SPL risk and compare with previous reports of RBC folate with different outcomes, we categorized parental RBC folate concentrations into ordinal variables (100 ng/mL intervals) and treated it as continuous in a GLM model to test the trend of associations ^8, 9^. To test the robustness of the primary analysis, we performed six sensitivity analyses. First, we used different definitions of SPL^1-3^ (Table S2). Second, we repeated the primary analysis models by excluding 561 fathers and 1025 mothers without blood samples (Figure 1). Third, to address potential residual confounding by socioeconomic status, we additionally adjusted for parental education level—a key factor that may influence both nutrient status (via diet quality and health literacy) and pregnancy outcomes, independently of lifestyle factors such as smoking or alcohol consumption. As SPL cases were mainly identified via hospital or city-level electronic medical records in this study, a non-trivial proportion of early pregnancy losses may not result in a hospital visit or may be managed in primary care settings not captured by the used systems, leading to potential under-ascertainment, especially of very early SPL cases. To address potential under ascertainment, especially of very early SPL cases that might not have led to a hospital visit, we performed an additional analysis in a sub cohort of 4,921 couples (enrolled after November 1^st^, 2018). In this sub-cohort, systematic telephone follow up was implemented specifically to capture SPL events that could have been missed by hospital, or city level electronic medical records. Furthermore, to explore whether the observed associations vary across well-established demographic and lifestyle strata, we conducted interaction and subgroup analyses for associations based on continuous parental RBC folate concentrations according to baseline age groups (<35 years vs. ≥35 years), preconception BMI categories (<24 kg/m² vs. ≥24 kg/m²), smoking exposures (yes vs. no), and alcohol consumption (yes vs. no). In addition, we validate primary analysis based on continuous nutrient levels at early gestation in a sub-cohort.

**4.3 Secondary aim analyses**

For the secondary aim analyses, we initially performed separate pairwise Pearson correlation analyses for mothers and fathers separately to explore cross correlations among metabolites. Next, we applied logistic LASSO regression ("glmnet" package) with SPL as the dependent variable and all measured metabolites as independent variables to identify metabolites with independent association with SPL. Ten-fold cross-validation was used to select the penalty term lambda(λ). We opted for λ.1se (the largest λ value within one standard error of the minimum deviance) to impose a stricter penalty than λ.min, thereby enhancing model parsimony. The standard errors of the LASSO coefficients were obtained via 1000 times bootstrapping. The λ.min and λ.1se for fathers and mothers were 0.02, 0.05 and 0.006, 0.07, respectively (Figure S13). To facilitate direct comparison of metabolite effects while maintaining biological interpretability, all metabolites were log-scaled to approximate normality and standardized to z-scores prior to analysis.

Missing data for exposures and covariates assumed as missing at random were imputed using multiple imputations with chained equations, and results were combined from the ten imputed datasets according to the Rubin rule^10^. Multiple testing was not adjusted in this study. All analyses were based on the imputed dataset.

**
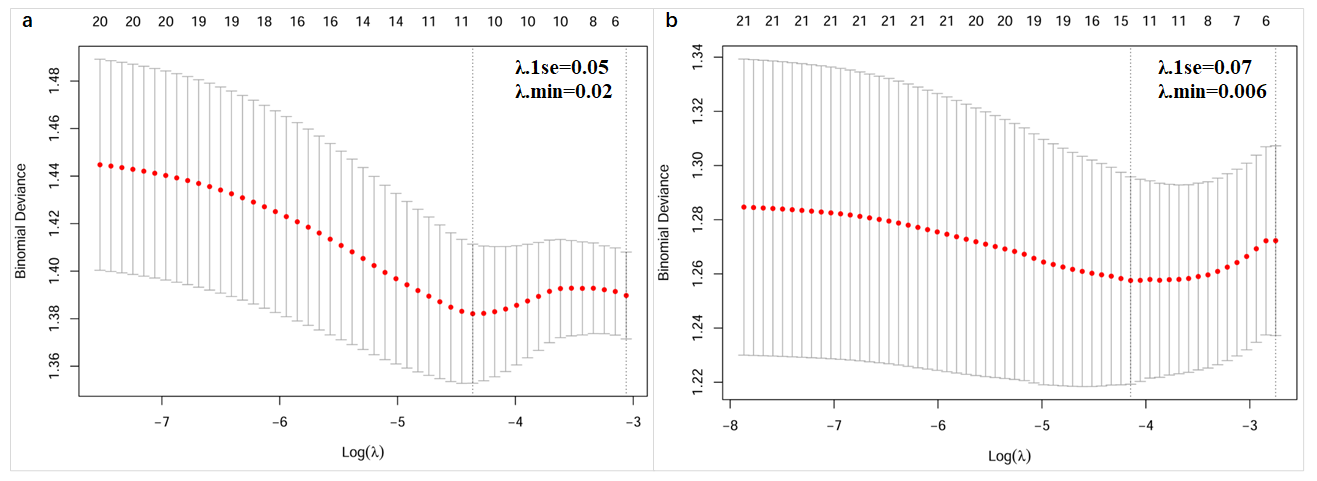
**

**Figure S15. Cross validation plot for the penalty term.** a, fathers; b, mothers. Age, preconception BMI, smoking and drinking status were treated as covariates, and preconception RBC folate and 16 OCM metabolites were treated as exposures in the LASSO regression model. To explore the associations among RBC folate, OCM metabolites, and SPL, we substituted 5-mTHF with RBC folate in the analysis, given that 5-mTHF constitutes the predominant metabolic component of RBC folate. The plot illustrates the relationship between the regularization parameter (λ) and the cross-validated mean squared error (MSE) for the LASSO regression model. Gray shaded region represents the±1 standard deviation of MSE across folds. Left dash line represent the optimal λ (λ.min) minimizing cross-validated MSE, and dash line represent the largest λ (λ.1se) within 1 standard error of the minimum MSE. Metabolites and RBC folate were normalized into z-scores prior to analysis.

**OCM Targeted Metabolomics in SPL**

**Participants**

The SPL cases and controls were derived from a Multiple Events Case-Control (MECC) study nested within the ongoing SPCC cohort. The MECC study comprises seven disease-case groups (including congenital heart disease, extreme/very preterm birth, SPL, Kawasaki disease, autism spectrum disorder, gestational diabetes, and preeclampsia) with a shared control group. Health controls were randomly matched to cases across seven disease groups based on maternal age, preconception BMI, and enrollment date. In this study, we included all 150 SPL cases (150 mothers and 147 fathers) and 200 controls (200 mothers and 173 fathers) from the MECC study (summarized in Table S5).

**Assay of OCM metabolites**

**Reagents**

Amino acid standards [glycine, L-serine, cystathionine, taurine, L-methionine, L-cysteine, oxidized L-glutathione (GSSG)] were purchased from Sigma-Aldrich (Sigma-Aldrich, Inc, USA). Choline standards [choline, betaine, dimethylglycine (DMG), and sarcosine] were purchased from Sigma-Aldrich (Sigma-Aldrich, Inc, USA). Additionally, deuterium-labeled internal standards (DMG-D3, Sarcosine-D3, Choline-D9, and Betaine-D3) were purchased from CDN (CDN Isotopes Inc., Canada). Finally, water-soluble vitamin standards including 4-Pyridoxic acid (VB6-PA), Pyridoxal 5'-phosphate (VB6-PLP), 5-Methyltetrahydrofolate (5-MeTHF), Methylmalonic acid (MMA) and 4 isotope internal standards (MMA-D3, VB6-PLP-D3, VB6-PA-D3, and 5-MeTHF-D3) were purchased from Sigma-Aldrich (Sigma-Aldrich, Inc., USA). Homocysteine (HCY), S-Adenosly Methionine (SAM), S-Adenosyl Homocysteine (SAH) were purchased from Sigma-Aldrich (Sigma-Aldrich, Inc, USA). Tetrahydrofolate (THF) was sourced from Schircks (Schircks Laboratories, Switzerland). Three isotope-labeled internal standards (HCY-D4, SAH-^13^C10, and FA-^13^C5) were purchased from CIL (Cambridge Isotope Laboratories, Inc., USA). SAM-^13^C5 were purchased from TRC (Toronto Research Chemicals, Canada). Analytical Reagent (AR) grade ascorbic acid, 5-amnoisoquinoline (5-AIQ), N-ethylmaleimide (NEM), and 4-tert-butylbenzenethiol (tBBT) were procured from Sigma-Aldrich (Sigma-Aldrich, Inc, USA). EDTA, tris(2-carboxyethyl) phosphine hydrochloride (TCEP) and N, N’-disuccinimidyl carbonate (DSC) were purchased from J&K (J&K Scientific, China). AR grade boric acid, Na2HPO4.12H2O and NaH2PO4.2H2O were acquired from SCRC (Sinopharm Chemical Reagent Co., Ltd., China). HPLC grade methanol and acetonitrile were sourced from Merck (Merck KGaA, Darmstadt, Germany), formic acid was purchased from Thermo Fisher (Thermo Fisher Scientific Inc., USA), dimethylsulfoxide (DMSO) was purchased from Tedia (Tedia, USA). European Pharmacopeia grade ammonium formate was purchased from Sigma-Aldrich (Sigma-Aldrich, Inc, USA).

**Metabolites extraction**

Amino acid extraction

Samples were thawed at 4 ℃ and 20 μL of serum were extracted from each sample. Then serum mixed with 60 μL of pre-cooled methanol to precipitate protein. The mixture was vortexed for 1 minute and centrifuged at 12000 rpm for 10 min at 4℃. A total of 10 μL of the supernatant was transferred and mixed with 10 μL of a solution (unspecified—please clarify if this is a buffer, solvent, etc.), followed by vortexing. Next, 10 μL of tBBT solution was added and mixed thoroughly. Then, 87 μL of borate buffer was added, and the mixture was vortexed for 30 seconds. Afterward, 33 μL of 5-AIQC solution was added, and the mixture was incubated at 55°C for 10 min. Following incubation, the mixture was cooled to ambient temperature, and 2 μL of formic acid was added. The solution was centrifuged again at 12,000 rpm for 10 min at 4°C. Finally, the supernatant was filtered through a 0.22 μm pore-size membrane and transferred into a sample vial for UPLC-MS/MS analysis.

Choline extraction

Internal standards composed of 6 μmol/L DMG-D3, 6 μmol/L Sarcosine-D3, 30 μmol/L Choline-D9, and 40 μmol/L Betaine-D3 in methanol. In total, 10μL serum samples, 5μL internal standards, and 485μLpre-cooled methanol were mixed and vortexed for 2 min. The mixture was centrifuged at 12000 rpm for 10 min at 4℃. Supernatant was transferred into sample vital for UPLC-MS/MS analysis.

Water-soluble vitamin extraction

Internal standards composed of 2000nmol/L MMA-D3, 1000 nmol/L VB6-PLP-D3, 600 nmol/L VB6-PA-D3, and 500 nmol/L 5-MeTHF-D3. A total of 50 μL of plasma sample, 5 μL of internal standard, and 200 μL of pre-cooled methanol (containing 0.05% VC and 0.5% formic acid) were vortexed and then centrifuged at 12,000 rpm for 10 min at 4°C. The supernatant was dried under nitrogen at ambient temperature and reconstituted in 100 μL of a methanol/water (1:9, v/v) solution. PH of the mixture was adjusted to 4.0 with NaOH solution and then centrifuged at 12000 rpm for 10 min at 4℃. Supernatant was transferred into sample vital for UPLC-MS/MS analysis.

Folate metabolites extraction

Internal standards composed of 40μmol/L HCY-D4, 0.8μmol/L SAM-^13^C5, 0.6 μmol/L SAH-^13^C10, 0.5 μmol/L FA-^13^C5 in methanol solution with methanol/water of 25/75 (v/v), containing 1%VC and 0.5% DTT. PH of the internal standards was adjusted to 7.0 with ammonium acetate. A total of 60 μL of serum, 6 μL of internal standard(s) (Note: use singular if one IS, plural if multiple), and 54 μL of methanol/water solution (25:75, v/v, containing 1% VC and 0.5% DTT) were vortexed for 30 sec and allowed to stand for 10 min at ambient temperature. Then, 240 μL of pre-cooled methanol was added to the mixture. The solution was vortexed for 2 min and centrifuged at 12,000 rpm for 10 min at 4°C. The supernatant was dried under a gentle stream of nitrogen at ambient temperature and reconstituted in 50 μL of methanol/water (25:75, v/v). The resulting solution was then filtered through a 0.22-μm membrane and transferred to an autosampler vial for UPLC-MS/MS analysis.

**UPLC-MS/MS conditions**

Instrument

The assay was performed with a UPLC-MS/MS consisting of an Agilent 1290 UPLC coupled to an Agilent 6470 triple-quadrupole MS equipped with an electrospray ionization (ESI) source (Agilent Technologies, USA).

Amino acid

Liquid chromatography was performed on Agilent ZORBAX Eclipse Plus C18 (2.1×100 mm, 1.8 μm particles). The temperature was set at 50 ℃ and 1μL of samples were injected on an UPLC column. Phase A and phase B were water and methanol containing 0.1% formic acid (v/v). The flow rate was 0.5mL/min. Mass spectrometric analysis was carried out in positive ion mode with electrospray ionization. Multiple reactions monitoring (MRM) was applied to quantify the screened fragment ions. Ion source condition was set as follows: the dry gas flow rate was 10L/min with temperature of 315℃, the pressure of nebulizer was 50 psi, and the sheath gas flow rate was 10L/min with temperature of 350℃, the nozzle voltage was set at 500V, and the capillary voltage was set at 4000V.

Choline

Liquid chromatography was performed on Waters ACQUITY UPLC BEH Amide (2.1×100 mm, 1.7 μm particles). The temperature was set at 40 ℃ and 1μL of samples were injected on an UPLC column. Phase A and phase B were 50% acetonitrile containing 0.1% formic acid (v/v) and 10Mm NH_4_COOH and 90% acetonitrile containing 0.1% formic acid (v/v) and 10Mm NH_4_COOH. The flow rate was 0.5mL/min. Mass spectrometric analysis was carried out in positive ion mode with electrospray ionization. Multiple reactions monitoring (MRM) was applied to quantify the screened fragment ions. Ion source condition was set as follows: the dry gas flow rate was 10L/min with temperature of 300℃, the pressure of nebulizer was 30 psi, and the sheath gas flow rate was 11L/min with temperature of 350℃, the nozzle voltage was set at 500V, and the capillary voltage was set at 4000V.

Water-soluble vitamin

Liquid chromatography was performed on Waters ACQUITY UPLC HSS T3 Column (2.1×100 mm, 1.8 µm particles). The temperature was set at 40 ℃ and 2μL of samples were injected on an UPLC column. Phase A and phase B were water containing 0.5% formic acid (v/v) and methanol containing 0.5% formic acid (v/v). The flow rate was 0.3mL/min. Mass spectrometric analysis was carried out in positive ion mode with electrospray ionization. Multiple reactions monitoring (MRM) was applied to quantify the screened fragment ions. Ion source condition was set as follows: the dry gas flow rate was 10L/min with temperature of 325℃, the pressure of nebulizer was 30 psi, and the sheath gas flow rate was 11L/min with temperature of 350℃, the nozzle voltage was set at 500V, and the capillary voltage was set at 4000V.

Folic acid

Liquid chromatography was performed on Agilent ZORBAX Eclipse Plus C18 (2.1×100 mm, 1.8 μm particles). The temperature was set at 50 ℃ and 1μL of samples were injected on an UPLC column. Phase A and phase B were water containing 0.1% formic acid (v/v) and methanol containing 0.1% formic acid (v/v). The flow rate was 0.4mL/min. Mass spectrometric analysis was carried out in positive ion mode with electrospray ionization. Multiple reactions monitoring (MRM) was applied to quantify the screened fragment ions. Ion source condition was set as follows: the dry gas flow rate was 10L/min with temperature of 315℃, the pressure of nebulizer was 30 psi, and the sheath gas flow rate was 11L/min with temperature of 350℃, the nozzle voltage was set at 500V, and the capillary voltage was set at 4000V.

**Quality control**

Quality control samples were prepared by mixing 15μL of each serum samples and injected before, during, and after the testing for 12 times. Additionally, six randomly selected parallel samples from the entire sample set were injected and tested three times to ensure accuracy and consistency.

**The Shanghai PreConception Cohort (SPCC) group**

Guoying Huang, Weili Yan, Xiaojing Ma, Weifen Luo, Wei Sheng, Yi Zhang, Yuan Jiang, Yin Ye, Dingmei Wang, Xiaotian Chen, Mengru Li, Hongyan Chen, Mi Ji, Yumei Liu, Gu Qing(s), Gu Qing(o), Linmei Zhu, De’ai Hou, Peiyu Sun, Xupeng Sun (Children’s Hospital of Fudan University, Shanghai, China); Hongbing Wang, Li Meng, Lin Zhang (Jingan Maternal and Child Health Center, Shanghai, China) ; Zifen Dai, Li fen (Shanghai First Maternity and Infant health Hospital, Shanghai, China); Shufang Chen, Zhenhua Tang, Jiahao Wu (International Peace Maternal and Child Health Hospital, Shanghai, China); Shuhua Wang, Dan li, Hui Wang (Xuhui Maternal and Child Health Center, Shanghai, China); Yu Ke, Weiping Cao, Baoren Zhang, Hong Huang (Shanghai Pudong New Area Health Care Hospital for Women & Children, Shanghai, China); Nailing Wang, Min Jiang, Jie Chen, Qiumin Xia (Shanghai Punan Hospital of Pudong New District, Shanghai, China); Hui Xu, Guoying Lao (Changning Maternity and Infant Health Hospital, Shanghai, China); HongMei Jin, Wenjuan Xie, Pin Yi (Qingpu Hospital, Zhongshan Hospital, Shanghai, China); Weiming Gong, JianXin Xu, Yingying Qian (Shanghai Qingpu Maternal and Child Health Center, Shanghai, China); Mingjie Luo, Jingwei Xia, Dongmei Chen, Zhenyu Tang (Shanghai Huangpu Maternal and Child Health Center, Shanghai, China); Xuejing Zhu, Qing Liu, Huiling Yang (Shanghai Huangpu Maternal and Child Health Hospital, Shanghai, China); Xiaotian Li, Zhiyong Wu, Chuanmin Ying, Shan Shi (Obstetrics and Gynecology Hospital of Fudan University (Shanghai Red House Obstetrics and Gynecology Hospital, Shanghai, China); Yanquan Zhang, Mingyi Yang (Wujing Hospital, Minhang District, Shanghai, Shanghai, China); Xiaohua Zhang, Lei Zhang, Lin Guan (Shanghai Minhang District Maternal and Child Health Care Hospital, Shanghai, China); Jinyu Xu, Honglin Wang, Fang Shen (The Fifth People's Hospital of Shanghai, Fudan University, Shanghai, China); Wenying Li, Xiaojing Teng, Jinling Zhao (Shanghai Minhang TCM Hospital, Shanghai, China); Cuili Zhu, Lan Wang, Hongwei Chen (Shanghai Songjiang District Central Hospital, Shanghai, China); Xiaoming Yuan, Meihua Zhang, Yaqiong Jin (Sijing Hospital, Songjiang District, Shanghai, China); Qing Yang, Wu Yan, Ying Wang, Hong Zhu, Min Feng (Songjiang Maternal and Child Health Center, Shanghai, China); Ying Wang, Yan Wu, Hong Tang (Songjiang Maternal and Child Health Hospital, Shanghai, China); Sa Guo (Tongji Hospital of Tongji University, Shanghai, China); Hongling Du (Shanghai Putuo District People's Hospital, Shanghai, China); Yuhuan Liu, Zhanyue Yi, Renhua Shi (Changhai Hospital, Second Military Medical University, Shanghai, Shanghai, China); Yu Gu, Qinfen Su, Yingying Lv (Shanghai Zhabei District Central Hospital, Shanghai, China); Yun Sun, Qiongpei Gu (Yangpu District Family Planning Service Center, Shanghai, China); Xixia Pang, Qingwu Zhang (Kong Jiang Hospital of Yangpu District, Shanghai, China); Songxiao Bai, Baoqiao Qi (Shanghai East City Hospital, Shanghai, China); Dong Junyin, Xie Rong, Gu Caihua, Zhang Xiangrong, He Hui, Shen Meifang, Tang Xiaxia, Zhang Shuanghu, Zhou Chengyan, Zhang Xiaoying, Yang Lili, Yao Hongping, Dai Sijia, Song Liujuan, Li Juan, Li Sibei, Wu Guirong, Gu Xiaowen, Gu Mingying, Qu Chunyan, Yan Bei, Wu Huijing, Tan Haiyun, Huang Ruonan, Shi Yongjie, Wang Zheng (Community Health Centers of Minhang District, Shanghai, China); Gu Xueyin, Zhou Ying, Zhu Weifang, Zou Hong, Lu Xinhua, Yao Huahui, Lin Xiangying, Zhang Hongwei, Wu Xiaofeng, Shen Ying, Ma Shengyan, Liang Xinxing, Wang Qing, Zhou Mingying, Ni Li, Ye Honglian, Li Jiayi, Gao Chunyan, Lu Xiuqin, Yin Qiuyi, Jin Jieping, Yan Lei, Meng Fanxin, Zhang Lan, Li Na, Chen Ru, Gu Zhufang, Tao Wenwei, Qiu Zhongfen, Gu Hongwei, Wu Weirong (Community Health Centers of Songjiang District, Shanghai, China).

**References**

1. Abortion GoCECoDaToS. Consensus of Chinese experts on diagnosis and treatment of spontaneous abortion (2020). *Chinese Journal of Practical Gynecology and Obstetrics*. 2020;**36**:1082-90.

2. RPL EGGo, Bender Atik R, Christiansen OB, et al. ESHRE guideline: recurrent pregnancy loss. *Hum Reprod Open*. 2018;**2018**:hoy004.

3. Ugurlu EN, Ozaksit G, Karaer A, et al. The value of vascular endothelial growth factor, pregnancy-associated plasma protein-A, and progesterone for early differentiation of ectopic pregnancies, normal intrauterine pregnancies, and spontaneous miscarriages. *Fertil Steril*. 2009;**91**:1657-61.

4. WHO. Manual of the international statistical classification of diseases, injuries, and causes of death : based on the recommendations of the ninth revision conference, 1975, and adopted by the Twenty-ninth World Health Assembly, 1975 revision. <https://apps.who.int/iris/handle/10665/40492> (accessed June 18, 2020).

5. McNutt LA, Wu C, Xue X, et al. Estimating the relative risk in cohort studies and clinical trials of common outcomes. *Am J Epidemiol*. 2003;**157**:940-3.

6. Hao L, Yang QH, Li Z, et al. Folate status and homocysteine response to folic acid doses and withdrawal among young Chinese women in a large-scale randomized double-blind trial. *Am J Clin Nutr*. 2008;**88**:448-57.

7. Cordero AM, Crider KS, Rogers LM, et al. Optimal serum and red blood cell folate concentrations in women of reproductive age for prevention of neural tube defects: World Health Organization guidelines. *MMWR Morb Mortal Wkly Rep*. 2015;**64**:421-3.

8. Daly LE, Kirke PN, Molloy A, et al. Folate levels and neural tube defects. Implications for prevention. *JAMA*. 1995;**274**:1698-702.

9. Chen HY, Zhang Y, Wang DM, et al. Periconception Red Blood Cell Folate and Offspring Congenital Heart Disease Nested Case-Control and Mendelian Randomization Studies. *Ann Intern Med*. 2022.

10. Sterne JA, White IR, Carlin JB, et al. Multiple imputation for missing data in epidemiological and clinical research: potential and pitfalls. *BMJ*. 2009;**338**:b2393.

STROBE Statement for the manuscript titled **"Preconception One-Carbon Metabolism Nutrient Levels in Preparing for Pregnancy Couples and Spontaneous Pregnancy Loss: A Prospective Cohort Study" (Manuscript ID: MCO2-2025-4748).**

|  | Item No | Recommendation | Page |
| --- | --- | --- | --- |
| **Title and abstract** | 1 | (*a*) Indicate the study’s design with a commonly used term in the title or the abstract | P1 |
|  |  | (*b*) Provide in the abstract an informative and balanced summary of what was done and what was found | P3 |
| Introduction | | |  |
| Background/rationale | 2 | Explain the scientific background and rationale for the investigation being reported | P4 |
| Objectives | 3 | State specific objectives, including any prespecified hypotheses | P5 |
| Methods | | |  |
| Study design | 4 | Present key elements of study design early in the paper | P13 |
| Setting | 5 | Describe the setting, locations, and relevant dates, including periods of recruitment, exposure, follow-up, and data collection | P13 |
| Participants | 6 | (*a*) *Cohort study*—Give the eligibility criteria, and the sources and methods of selection of participants. Describe methods of follow-up  *Case-control study*—Give the eligibility criteria, and the sources and methods of case ascertainment and control selection. Give the rationale for the choice of cases and controls  *Cross-sectional study*—Give the eligibility criteria, and the sources and methods of selection of participants | P13 |
|  |  | (*b*) *Cohort study*—For matched studies, give matching criteria and number of exposed and unexposed  *Case-control study*—For matched studies, give matching criteria and the number of controls per case | NA |
| Variables | 7 | Clearly define all outcomes, exposures, predictors, potential confounders, and effect modifiers. Give diagnostic criteria, if applicable | P13-14 |
| Data sources/ measurement | 8* | For each variable of interest, give sources of data and details of methods of assessment (measurement). Describe comparability of assessment methods if there is more than one group | P14 |
| Bias | 9 | Describe any efforts to address potential sources of bias | P15 |
| Study size | 10 | Explain how the study size was arrived at | NA |
| Quantitative variables | 11 | Explain how quantitative variables were handled in the analyses. If applicable, describe which groupings were chosen and why | P14-15 |
| Statistical methods | 12 | (*a*) Describe all statistical methods, including those used to control for confounding | 15, and supplementary file P24-25 |
|  |  | (*b*) Describe any methods used to examine subgroups and interactions | 15, and supplementary file P25 |
|  |  | (*c*) Explain how missing data were addressed | Supplementary file P25 |
|  |  | (*d*) *Cohort study*—If applicable, explain how loss to follow-up was addressed  *Case-control study*—If applicable, explain how matching of cases and controls was addressed  *Cross-sectional study*—If applicable, describe analytical methods taking account of sampling strategy | P15 |
|  |  | (*e*) Describe any sensitivity analyses | P15, and supplementary file P25 |

Continued on next page

| Results | | |  |
| --- | --- | --- | --- |
| Participants | 13* | (a) Report numbers of individuals at each stage of study—eg numbers potentially eligible, examined for eligibility, confirmed eligible, included in the study, completing follow-up, and analysed | P5 |
|  |  | (b) Give reasons for non-participation at each stage | P5 |
|  |  | (c) Consider use of a flow diagram | P5 |
| Descriptive data | 14* | (a) Give characteristics of study participants (eg demographic, clinical, social) and information on exposures and potential confounders | P5 |
|  |  | (b) Indicate number of participants with missing data for each variable of interest | NA |
|  |  | (c) *Cohort study*—Summarise follow-up time (eg, average and total amount) | NA |
| Outcome data | 15* | *Cohort study*—Report numbers of outcome events or summary measures over time | P5 |
|  |  | *Case-control study—*Report numbers in each exposure category, or summary measures of exposure | NA |
|  |  | *Cross-sectional study—*Report numbers of outcome events or summary measures | NA |
| Main results | 16 | (*a*) Give unadjusted estimates and, if applicable, confounder-adjusted estimates and their precision (eg, 95% confidence interval). Make clear which confounders were adjusted for and why they were included | P7 |
|  |  | (*b*) Report category boundaries when continuous variables were categorized | P7 |
|  |  | (*c*) If relevant, consider translating estimates of relative risk into absolute risk for a meaningful time period | NA |
| Other analyses | 17 | Report other analyses done—eg analyses of subgroups and interactions, and sensitivity analyses | P7-8 |
| Discussion | | |  |
| Key results | 18 | Summarise key results with reference to study objectives | P8-9 |
| Limitations | 19 | Discuss limitations of the study, taking into account sources of potential bias or imprecision. Discuss both direction and magnitude of any potential bias | P10-12 |
| Interpretation | 20 | Give a cautious overall interpretation of results considering objectives, limitations, multiplicity of analyses, results from similar studies, and other relevant evidence | P11-12 |
| Generalisability | 21 | Discuss the generalisability (external validity) of the study results | P12 |
| Other information | | |  |
| Funding | 22 | Give the source of funding and the role of the funders for the present study and, if applicable, for the original study on which the present article is based | P17 |

*Give information separately for cases and controls in case-control studies and, if applicable, for exposed and unexposed groups in cohort and cross-sectional studies.

**Note:** An Explanation and Elaboration article discusses each checklist item and gives methodological background and published examples of transparent reporting. The STROBE checklist is best used in conjunction with this article (freely available on the Web sites of PLoS Medicine at http://www.plosmedicine.org/, Annals of Internal Medicine at http://www.annals.org/, and Epidemiology at http://www.epidem.com/). Information on the STROBE Initiative is available at www.strobe-statement.org.
